# HPV16 Down-Regulates the Insulin-Like Growth Factor Binding Protein 2 to Promote Epithelial Invasion in Organotypic Cultures

**DOI:** 10.1371/journal.ppat.1004988

**Published:** 2015-06-24

**Authors:** Adam Pickard, Simon S. McDade, Marie McFarland, W. Glenn McCluggage, Cosette M. Wheeler, Dennis J. McCance

**Affiliations:** 1 Centre for Cancer Research and Cell Biology, Queen’s University Belfast, Belfast, United Kingdom; 2 Belfast Health and Social Care Trust, Belfast, United Kingdom; 3 Department of Pathology, School of Medicine, University of New Mexico, Albuquerque, New Mexico, United States of America; University of Wisconsin-Madison, UNITED STATES

## Abstract

Cervical cancer is a multi-stage disease caused by human papillomaviruses (HPV) infection of cervical epithelial cells, but the mechanisms regulating disease progression are not clearly defined. Using 3-dimensional organotypic cultures, we demonstrate that HPV16 E6 and E7 proteins alter the secretome of primary human keratinocytes resulting in local epithelial invasion. Mechanistically, absence of the IGF-binding protein 2 (IGFBP2) caused increases in IGFI/II signalling and through crosstalk with KGF/FGFR2b/AKT, cell invasion. Repression of IGFBP2 is mediated by histone deacetylation at the IGFBP2 promoter and was reversed by treatment with histone deacetylase (HDAC) inhibitors. Our *in vitro* findings were confirmed in 50 invasive cancers and 79 cervical intra-epithelial neoplastic lesions caused by HPV16 infection, where IGFBP2 levels were reduced with increasing disease severity. In summary, the loss of IGFBP2 is associated with progression of premalignant disease, and sensitises cells to pro-invasive IGF signalling, and together with stromal derived factors promotes epithelial invasion.

## Author Summary

The human papillomaviruses (HPV) are the etiological agents of cervical cancer and the disease progresses through the pre-malignant phases of cervical intraepithelial neoplasia I, II and III (CINI-III), before becoming an invasive carcinoma. Therefore identifying factors, which regulate the transition through the premalignant phases and onto invasive cancer would be of importance clinically, to identify patients at risk of progressing from CIN I to CIN III. We show that expression of E6 and E7 proteins from the high risk HPV16, causes reduced expression of the IGF binding protein 2 (IGFBP2) and this correlates with progression from CIN I to CIN III. By modulating IGFBP2 levels in epithelial cells, we have demonstrated that reduction of IGFBP2 levels is a driving event in epithelial invasion. We have gone on to show that de-regulation of expression of IGFBP2 is due to histone deacetylation of the promoter, which can be reversed by histone deacetylase inhibitors.

## Introduction

Metastasis involves multiple steps, so defining the processes which regulate cancer cell invasion are crucial for understanding the initiation of the metastatic process. In particular, it will be important to monitor the molecular events that occur in the transition from a hyper-proliferative epithelium to an invasive epithelium and determine their functions. High-risk human papillomavirus (HPV) types are responsible for the transformation of the cervical epithelium and subsequent cervical cancer. Expression of the ‘early’ HPV genes E6 and E7 has been identified to be sufficient to immortalise primary human keratinocytes [1,2] and are required for continued proliferation of infected cells however whether this is sufficient to transform cells into a malignant form is still disputed [2–4]. E6 and E7 proteins immortalize epithelial cells through their ability to inactivate the cell cycle checkpoints regulated by the retinoblastoma protein (pRb) and p53 resulting in enhanced proliferation and loss of differentiation [5–7]. If not cleared, the HPV infection can persist resulting in progression to invasive disease [8]. However, not all HPV infections of the cervix lead to progressive disease and so knowledge of the alterations during transition from low grade, CIN 1, to high grade disease, CIN 3 and eventual invasive disease may yield novel molecular biomarkers that distinguish lesions with a propensity to progress to invasive disease from lesions that will remain premalignant [9].

During the development of cervical cancer, numerous molecular events have been described, including: altered viral gene expression [10,11], regulation of immune-response [12], activation of proliferative signalling pathways [13–15], modification of chromatin [16–19], and regulation of pro-invasive genes, such as matrix metalloproteases (MMPs) [11,20]. In the present study, we have investigated the factors and mechanisms which influence the invasive behaviour of the epithelium. We have examined the ability of the high-risk HPV16 E6 and E7 genes to transform primary human foreskin keratinocytes (HFKs) into an invasive epithelium and have identified a crucial role for the IGF (Insulin-like growth factor) signalling pathway in the progression to invasive growth. The invasive potential of E6/7 expressing keratinocytes is enhanced following dramatic down-regulation of insulin-like growth factor binding protein 2 (IGFBP2), resulting from enhanced histone deacetylase 3 activity at the IGFBP2 promoter. IGFBP2 has been shown to have both pro-tumourgenic properties and tumour suppressive functions, although the former tend to be independent of IGF/IGF receptor signalling [21]. In this study, we have found that IGFBP2 acts to suppress IGFI/II stimulation of the IGF receptor 1 (IGF1R) but in its absence, IGFI/II signalling, in conjunction with the stromal derived growth factor, keratinocyte growth factor (KGF), stimulates the AKT pathway leading to invasion. Significantly, we have observed that IGFBP2 expression is inhibited in high-grade pre-malignant cervical lesions infected with HPV16 and propose that this down-regulation is a required step in the initiation of the invasion process.

## Results

**Fig 1:**
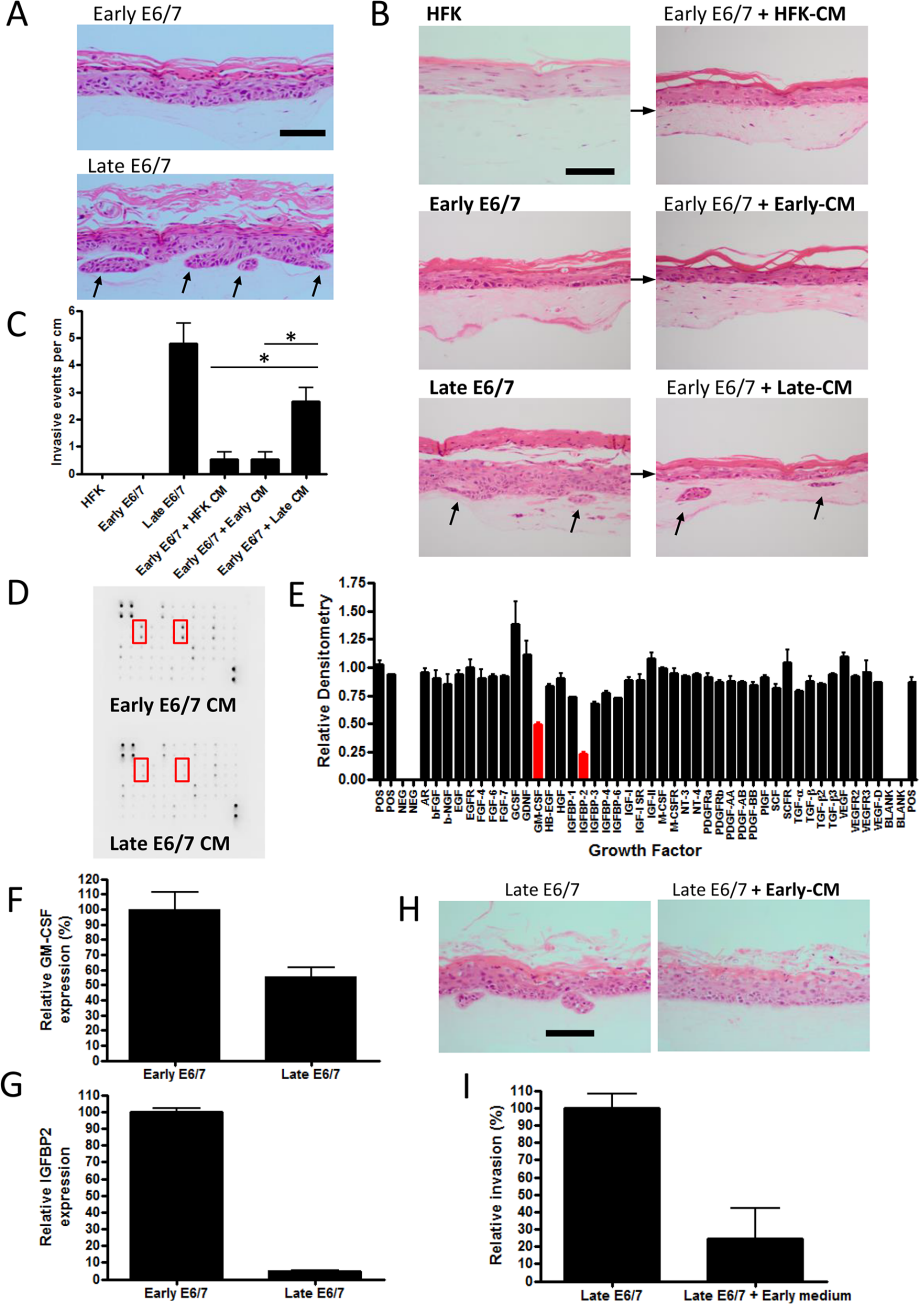
Prolonged passage of HPV16 E6/7 immortalised keratinocytes generates an invasive epithelium. A) H+E stained sections of organotypic cultures containing early and late passage E6/7-HFKs cultured with pro-invasive pRb-depleted fibroblasts. Late-passage but not early passage E6/7-HFKs invade into the collagen matrix. B) Medium transfer from late-passage E6/7-HFKs (Late-CM) can promote invasion in early pass E6/7-HFKs unlike medium from normal HFKs (HFK-CM) and early-passage E6/7-HFKs (Early-CM). The frequency of invasion is shown in C), data represents the average of three independent experiments, error bars represent standard error of the means (SEM). CM = conditioned medium. D) Growth factor array analysis of conditioned medium, which is quantified in E) demonstrates late-passage E6/7-HFKs secrete less IGFBP2 and GM-CSF. F and G) GM-CSF and IGFBP2 mRNA transcript levels are reduced in late-passage E6/7-HFKs compared to non-invasive early pass E6/7-HFKs. n = 3, error bars represent SEM. H) Medium transfer from non-invasive early E6/7-HFKs blocks invasion of late-passage E6/7-HFK cultures as quantified in I). n = 3, error bars represent SEM. Scale bars represent 100 μM.

The high-risk HPVs are a causal factor for cervical and infection is observed in a proportion of head and neck cancers. Whilst E6 and E7 expressing keratinocytes are immortalized, we have observed in three-dimensional organotypic cultures that they do not possess the ability to invade into the stroma [1,2]. The stromal compartment also regulates the invasive behaviour of the epithelium [22–24], and we have recently demonstrated that pRb-depleted human foreskin fibroblasts (HFFs) promote epithelial invasion, i.e. breakdown of the basement membrane and growth into the underlying collagen layer (S1 Fig). This invasion is driven through altered secretion of the keratinocyte growth factor [22]. Organotypic cultures generated using early-passage (passage 3–10) HPV16 E6/7 expressing HFKs are refractory to the pro-invasive signals from pRb-depleted HFFs. However, with continued passage (late passage) (i.e. >passage 14), these cells acquire the ability to invade (Fig 1A). We hypothesized that following extended passage. E6/7-HFKs may secrete growth factors or cytokines that could alter invasive behaviour of the epithelium.

To test this hypothesis, conditioned medium (CM) from monolayer cultures of invasive cells, normal HFKs and early-passage E6/7-HFKs was transferred daily to organotypic cultures containing non-invasive early E6/7-HFKs. Medium taken from late-passage E6/7-HFKs was sufficient to induce invasion of the previously non-invasive cells, although not to the same level as late passage E6/7-HFKs (Fig 1B and 1C), suggesting invasive late-passage E6/7-HFKs were generating a pro-invasive environment. Subsequently, conditioned medium from normalised numbers of early and late-passage E6/7-HFKs were subjected to growth factor array analysis, which measured the levels of 41 growth factors. Surprisingly, the pro-invasive late-passage E6/7-HFKs did not secrete additional growth factors/cytokines but produced significantly lower levels of the insulin-like growth factor binding protein, IGFBP2, and the granulocyte-macrophage colony-stimulating factor (GM-CSF) at the protein (Fig 1D and 1E) and mRNA level (Fig 1F and 1G) in cell extracts. This result suggested that an inhibitor of invasion was lost as cells acquired invasive behaviour, indeed, when media from non-invasive, early pass E6/7-HFKs was transferred to invasive late-passage E6/7-HFKs, we observed a complete inhibition of invasion (Fig 1H and 1I).

**Fig 2:**
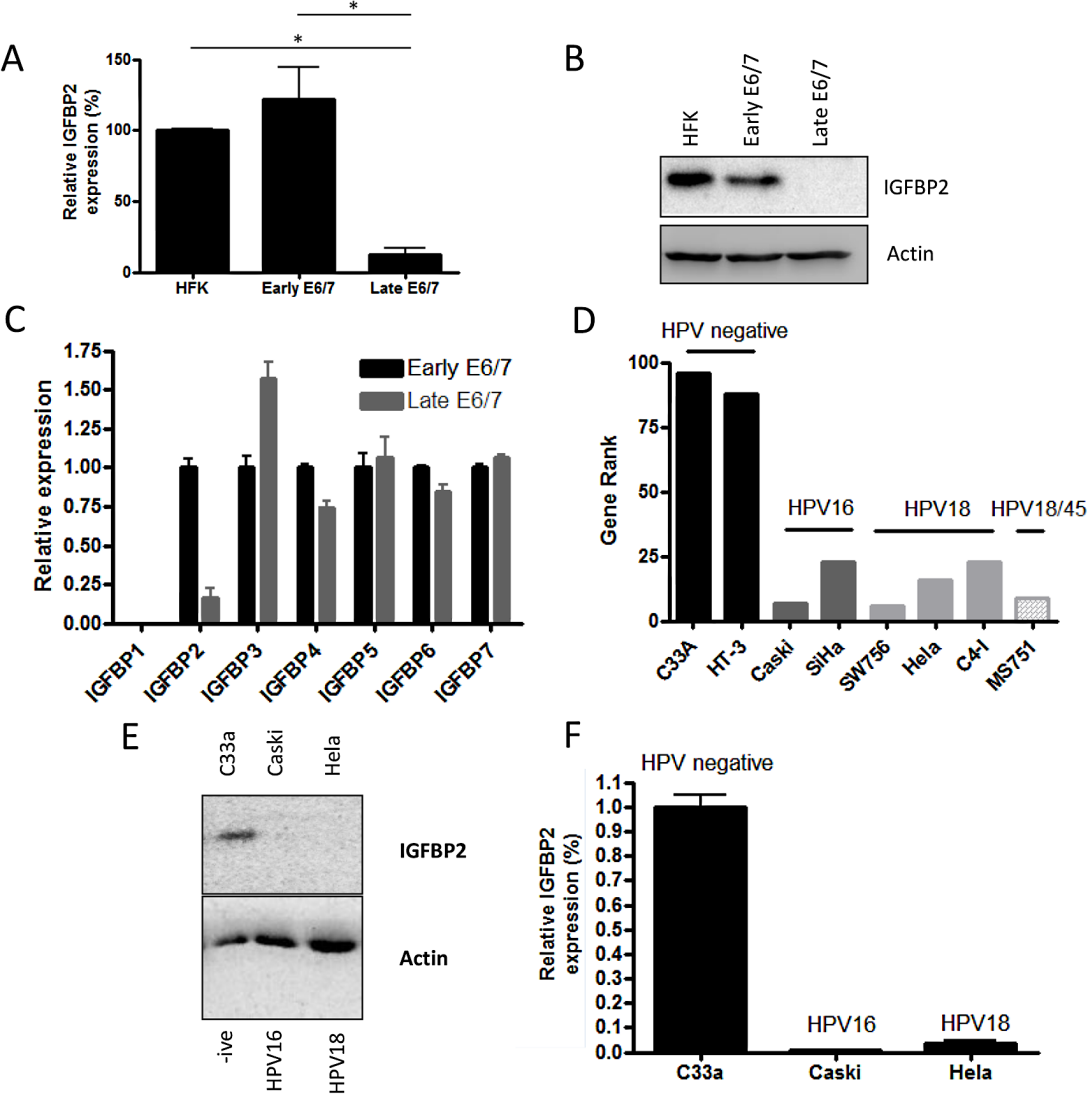
IGFBP2 is transcriptionally repressed in late-passage E6/7-HFKs and HPV positive cervical cancer cell lines. A) Continued expression of E6/7 in HFKs leads to dramatic down-regulation of IGFBP2 mRNA levels, n = 4, error bars represent SEM, this is also observed at a protein level (B). C) Real-time PCR analysis of the IGFBP family of proteins demonstrated a specific down regulation of IGFBP2 expression. n = 3, error bars represent SEM. Note IGFBP1 is not expressed by HFKs but was readily detected in human fibroblasts (S4A Fig). D) Gene rank analysis of IGFBP2 expression in cervical cells lines identified that IGFBP2 expression is low in HPV positive cell lines in comparison to cervical HPV negative cell lines (C33A and HT-3). E) The results in D were confirmed by western blot (E) and real-time PCR analysis (F) for the C33a, Caski and Hela cervical cell lines. n = 3, error bars represent SEM.

Since IGFBP2 expression was the most dramatically altered factor (>90% loss, p<0.001), we wanted to investigate its role in the invasive phenotype of late passage E6/7-HFKs. Western blot and real-time analysis from three independently generated E6/7-HFK lines confirmed that late-passage E6/7-HFKs produced very low levels of IGFBP2 in comparison to early-passage E6/7-HFKs as well as primary HFKs (Fig 2A and 2B, and S2A Fig). Down-regulation of IGFBP2 also occurred with continued passage of E6/7-HFKs maintained in co-culture with J2-3T3 fibroblasts in E-medium (S2B Fig). Further examination of the IGFBP family identified that there was a modest increase in IGFBP3 but only IGFBP2 was significantly regulated following continued passage of E6/7-HFKs (Fig 2C). These results implied that IGFBP2 may be down-regulated either as a consequence of long term culture or prolonged HPV16 E6/7 expression. The expression of IGFBP2 was therefore monitored in primary human foreskin keratinocytes and immortalised keratinocytes (immortalised either by hTERT or through co-culture with J2-3T3 mouse fibroblasts and the Rock inhibitor Y27632 [23]). IGFBP2 was expressed at high levels in immortalized cells relative to late passage E6/7 keratinocytes (S2C and S2D Fig). Furthermore, we established that HPV16 E6/7 were able to mediate down-regulation of IGFBP2 when introduced into these immortalised cells whereas control cells transfected with vector only did not (S2E and S2F Fig). This implied that the down-regulation of IGFBP2 was a result of prolonged HPV16 E6/7 expression. To test this we targeted E6 and E7 in late passage E6/7-HFKs with siRNA, which resulted in re-expression of p53, pRb, and IGFBP2 and concomitantly inhibited invasion in organotypic cultures (S3A–S3D Fig). There is also a correlation between IGFBP2 levels and HPV in publicly available microarray datasets from various cervical cancer cell lines [24] where HPV positive cervical cancer cell lines were shown to have substantially reduced IGFBP2 expression compared to HPV negative cervical cell lines (Fig 2D). We independently confirmed this at protein and RNA levels in C33a, Caski and Hela cell lines (Fig 2E and 2F).

**Fig 3:**
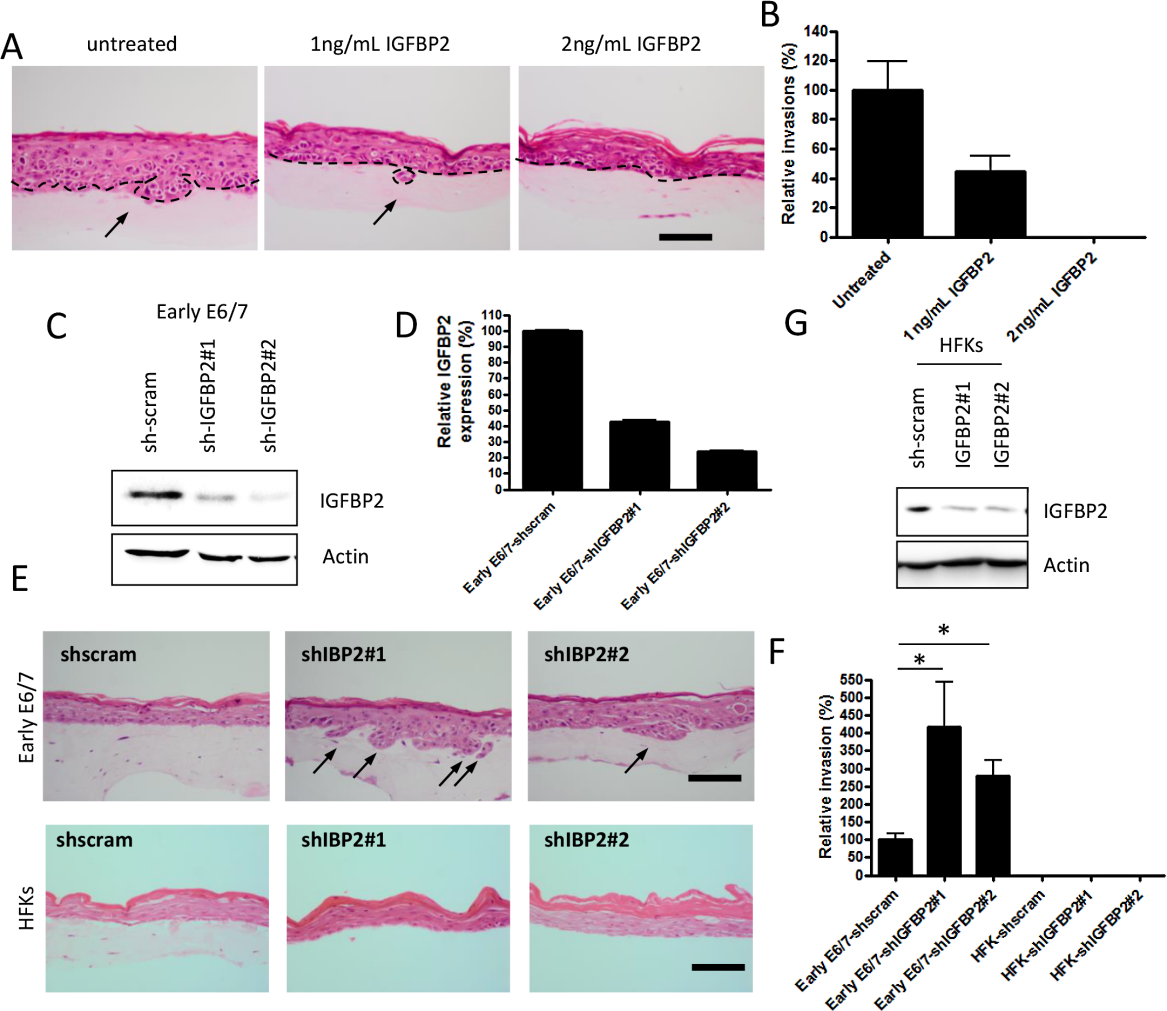
IGFBP2 is a regulator of the invasion process. A) Recombinant IGFBP2 was added to organotypic cultures at the indicated concentrations over a 14 day period. Addition of IGFBP2 inhibited the invasive behaviour of late-passage E6/7-HFKs and the invasive frequencies are quantified in B). n = 3, error bars represent SEM. C) IGFBP2 levels were depleted from early-passage E6/7-HFKs as demonstrated by western blot (C) and real-time analysis (D). n = 3, error bars represent SEM. E) Depletion of IGFBP2 from early-passage E6/7-HFKs resulted in enhanced invasive potential as quantified in F). However this was not observed when IGFBP2 was depleted in primary HFKs (G, also E and F). n = 3, error bars represent SEM. Scale bars represent 100 μM.

To establish a role for IGFBP2 in the invasion process, recombinant IGFBP2 was added to organotypic cultures containing invasive late-passage E6/7-HFKs. Addition of physiological quantities of IGFBP2 to the cultures resulted in inhibition of epithelial invasion in a dose dependent manner (Fig 3A and 3B, and S4B and S4C Fig). As IGFBP3 was found to be elevated in late passage cultures and is thought to promote invasion, we assessed whether IGFBP3 addition further induced epithelial invasion in late passage E6/7-HFKs (S4D and S4E Fig), but found no further effect of IGFBP3 addition. This was also established in a modified organotypic raft culture containing the HPV16 positive cervical cancer cell line, Caski (S4F and S4G Fig), where IGFBP2, but not IGFBP3, significantly inhibited invasion of the epithelial cells. To further assess the effects of IGFBP2 loss on the invasive potential of the epithelium, IGFBP2 levels were stably depleted in early-passage non-invasive E6/7-HFKs using two different shRNA molecules. IGFBP2 knockdown was confirmed by Western blot and real-time PCR analysis (Fig 3C and 3D), and resulted in enhanced invasion (Fig 3E and 3F). IGFBP2 was also depleted in primary HFKs (Fig 3G), however, this did not result in the generation of an invasive epithelium (Fig 3E and 3F) suggesting that IGFBP2 acts as a brake to pro-invasive signalling mediated by E6 and E7 and following continued cell expansion, this brake is lost.

**Fig 4:**
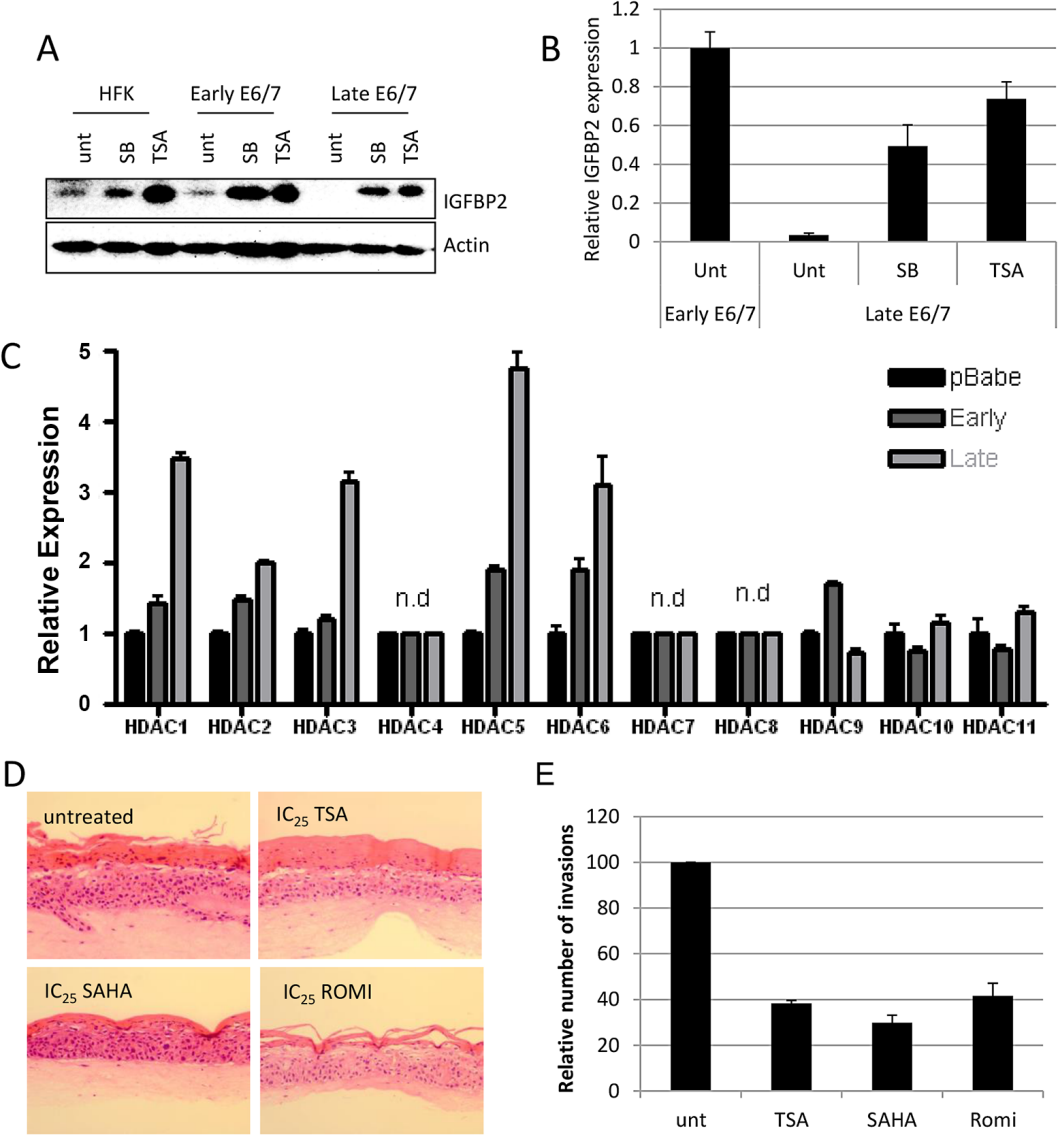
IGFBP2 expression is repressed through enhanced HDAC function. A) Addition of 1 μM Trichostatin A (TSA) or 5 mM sodium butyrate (SB) restores expression of IGFBP2 in late passage E6/7-HFKs to levels similar to primary keratinocytes, as demonstrated by Western blot and real-time PCR (B). n = 3, error bars represent SEM. C) The expression levels of histone deacetylases were assessed by real-time PCR in control primary keratinocytes (pBabe) early and late passage E6/7-HFKs. n = 3, representative experiment shown, error bars represent standard deviation (SD). D) Addition of HDAC inhibitors to organotypic cultures significantly reduced the invasive frequency of the epithelium, as quantified in (E). n = 3, error bars represent SEM.

IGFBP2 expression is regulated by a variety of factors, including the IGF system itself [25] and insulin [26], however in E6/7 expressing keratinocytes, we found this was not the case (S4H Fig). Epigenetic mechanisms have been associated with regulation IGFBP2 expression [27–29], so next we wanted to determine if one or more of these mechanisms played a role in IGFBP2 regulation and if manipulation of the epigenetic factors would restore the IGFBP2 levels in late pass E6/7-HFKs. We have identified that as E6/7 immortalised keratinocytes are passaged, there is acquisition of methylation marks at CpG islands close to the transcriptional start of IGFBP2 in late passage cells only (S5A and S5B Fig). However addition of 5-aza-C was unable to restore expression of IGFBP2 in the invasive cells (S5C and S5D Fig) suggesting DNA methylation may not be the critical regulator of IGFBP2. HDAC inhibitors have been shown to elevate expression of IGFBP2 in cells which readily express the protein [28,29], so to determine if this had occurred in late passage cells, which exhibit low levels of IGFBP2, we added the pan-HDAC inhibitors sodium butyrate (SB) or trichostatin A (TSA) to invasive E6/7-expressing keratinocytes and Hela cells. The inhibitors restored both IGFBP2 mRNA and protein (Fig 4A and 4B) in late E6/E7 keratinocytes, as well as in Hela cells (S6 Fig). Since these results suggest that there is increased histone deacetylation in the invasive keratinocytes resulting in reduced expression of IGFBP2, we determined the expression of HDACs during the transition to an invasive epithelium. HDAC 1, 2, 3, 5 and 6 were elevated in the invasive epithelial cells (Fig 4C), and cell survival assays suggested that these invasive cells are sensitive to HDAC inhibition (S7 Fig). Interestingly, addition of low doses (IC_25_) of the HDAC inhibitors TSA, SAHA (both pan inhibitors) and romidepsin (Romi, a class I inhibitor, which inhibits HDACs 1, 2, 3 but also HDAC4 and 6), which do not inhibit proliferation, was sufficient to reduce invasive frequency of the late passage E6/7-HFKs (Fig 4D and 4E).

**Fig 5:**
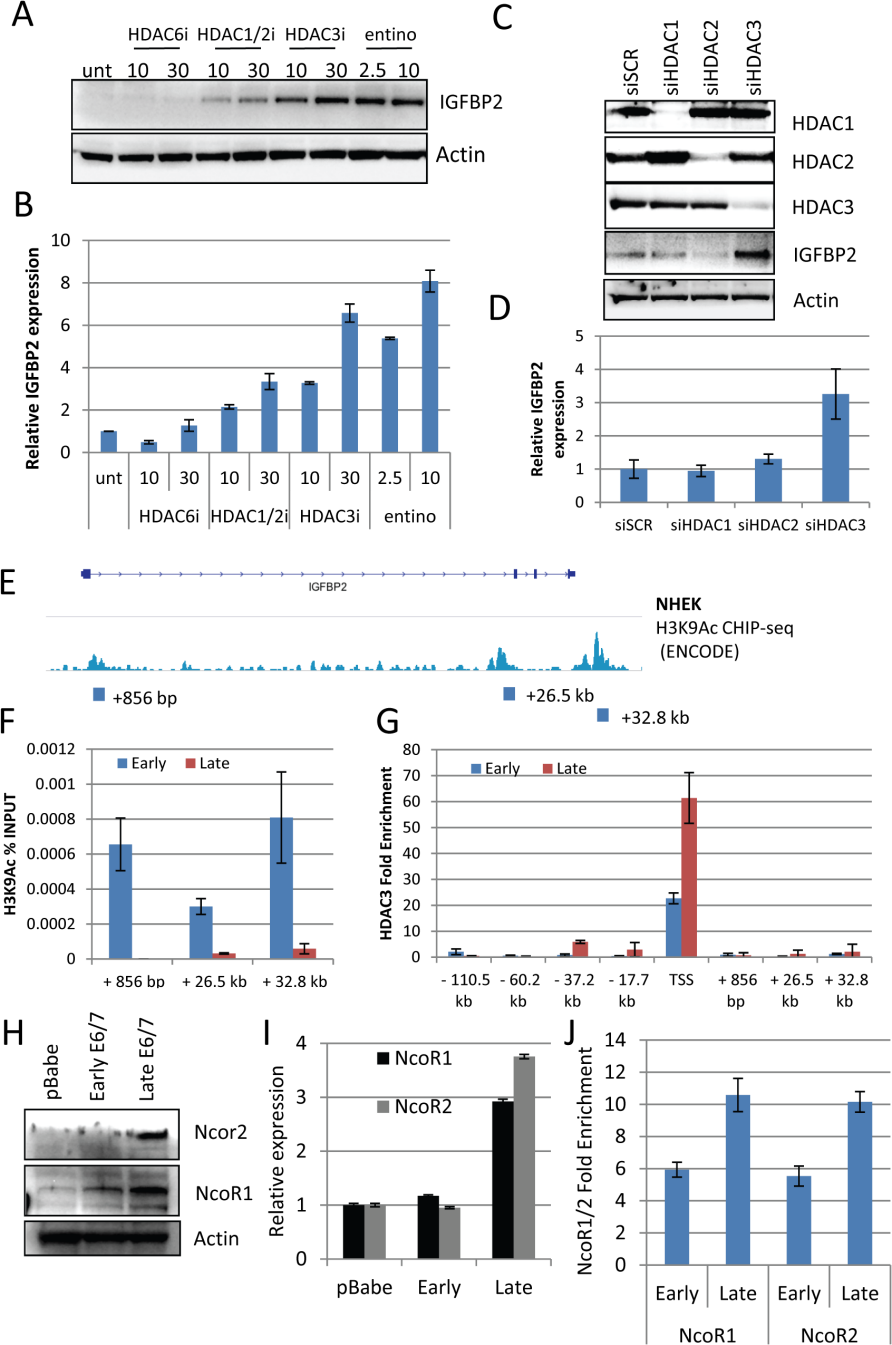
HDAC3 is a critical regulator of IGFBP2 expression. A) Using selective (HDAC6i, HDAC1/2i and HDAC3i) HDAC inhibitors and a class I inhibitor (entinostat – entino), we were able to restore IGFBP2 expression in late passage E6/7-HFKs both at the protein and transcriptional level (B). n = 3, representative experiment shown, error bars represent SD. C) Following 48 hours treatment with siRNA targeting HDAC1, 2 or 3, western blot analysis confirmed the specific depletion of individual HDACs siRNA and restoration of IGFBP2 following HDAC3 depletion. D) This was also confirmed at the transcriptional level n = 3, error bars represent SEM. E) Encode histone 3 lysine 9 acetylation (H3K9Ac) CHIP-seq from normal human epithelial keratinocytes (NHEK) at the IGFBP2 locus. Blocks represent primer locations to assess the levels of H3K9Ac binding at these sites. F) H3K9Ac CHIP-qPCR at the three sites identified in E, demonstrates the loss of the modification in late passage E6/7-HFKs which lack IGFBP2 expression. n = 3, error bars represent SEM G) HDAC3 CHIP-qPCR was conducted at locations throughout the IGFBP2 locus, predicted from previous HDAC3 CHIP-seq experiments in human and mouse derived cells. HDAC3 binding was enriched at the transcriptional start site (TSS) and was enhanced in late passage E6/7-HFKs. n = 3, error bars represent SEM. H) Western blot of NcoR1 and NcoR2 in HFKs, early and late passage E6/7-HFKs. I) The co-repressors NcoR1 and NcoR2 are elevated in late passage E6/7 cells at the protein and transcriptional level (I). n = 3, error bars represent SEM J) CHIP-qPCR analysis also demonstrated enhanced binding of both NcoR1 and NcoR2 at the TSS. Average of two experiments shown, error bars represent SD.

To further evaluate the mechanism through which IGFBP2 expression is regulated by histone modifications, selective HDAC inhibitors (proprietary inhibitors of HDAC6 (HDAC6i), HDAC1 and 2 (HDAC1/2i) and HDAC3 (HDAC3i)) and entinostat, which is another class I inhibitor, were employed. Inhibitors of HDAC3, the class I inhibitor, entinostat and to a lesser extent the HDAC1/2 inhibitor were sufficient to restore IGFBP2 RNA and protein expression (Fig 5A and 5B). Using a commercially available HDAC3 selective inhibitor, RGFP966, IGFBP2 expression was also restored (S8 Fig). To further confirm a specific role for HDAC3 in the regulation of IGFBP2 expression, HDAC1-3 were individually depleted using siRNA and results showed that depletion of HDAC3 was sufficient to restore both mRNA and protein expression of IGFBP2 (Fig 5C and 5D).

To assess the function of HDAC3 in regulating IGFBP2 expression we utilised publicly available data, which examined histone modifications around the IGFBP2 locus in primary keratinocytes [30] (Fig 5E). Three histone 3 lysine 9 (H3K9) acetylation sites, which can be altered by HDAC3 [31], were identified. Using CHIP-qPCR with anti-H3K9Ac CHIP-tested antibodies [30], we observed that the acetylation at these three sites is lost in invasive cells, which do not express IGFBP2 (Fig 5F), suggesting a mechanism for the loss of expression of IGFBP2 in late passage cells. In addition, the IGFBP2 promoter is bivalent, containing both active and repressive histone modifications within the promoter region, with the activating modifications proposed to predominate over the repressive elements [32]. Our results suggest that the loss of the activating marks allows the repressive elements to predominate leading to repression of the gene expression as previously suggested [33]. To support this suggestion, we showed, using ChIP-qPCR, that there is an increase in the presence of HDAC3 at the transcriptional start site, but no significant enrichment of HDAC3 at other sites within the IGFBP2 locus (Fig 5G). Furthermore, HDAC3 resides in a repressive complex with NcoR1 (NcoR1) and NcoR2 (SMRT) [34] resulting in an active repressive complex [35]. Analysis of NcoR1/2 at the protein and transcriptional levels showed marked elevation in invasive cells (Fig 5H and 5I) and also an enrichment at the Transcriptional Start Site (TSS) of IGFBP2 (Fig 5J).

**Fig 6:**
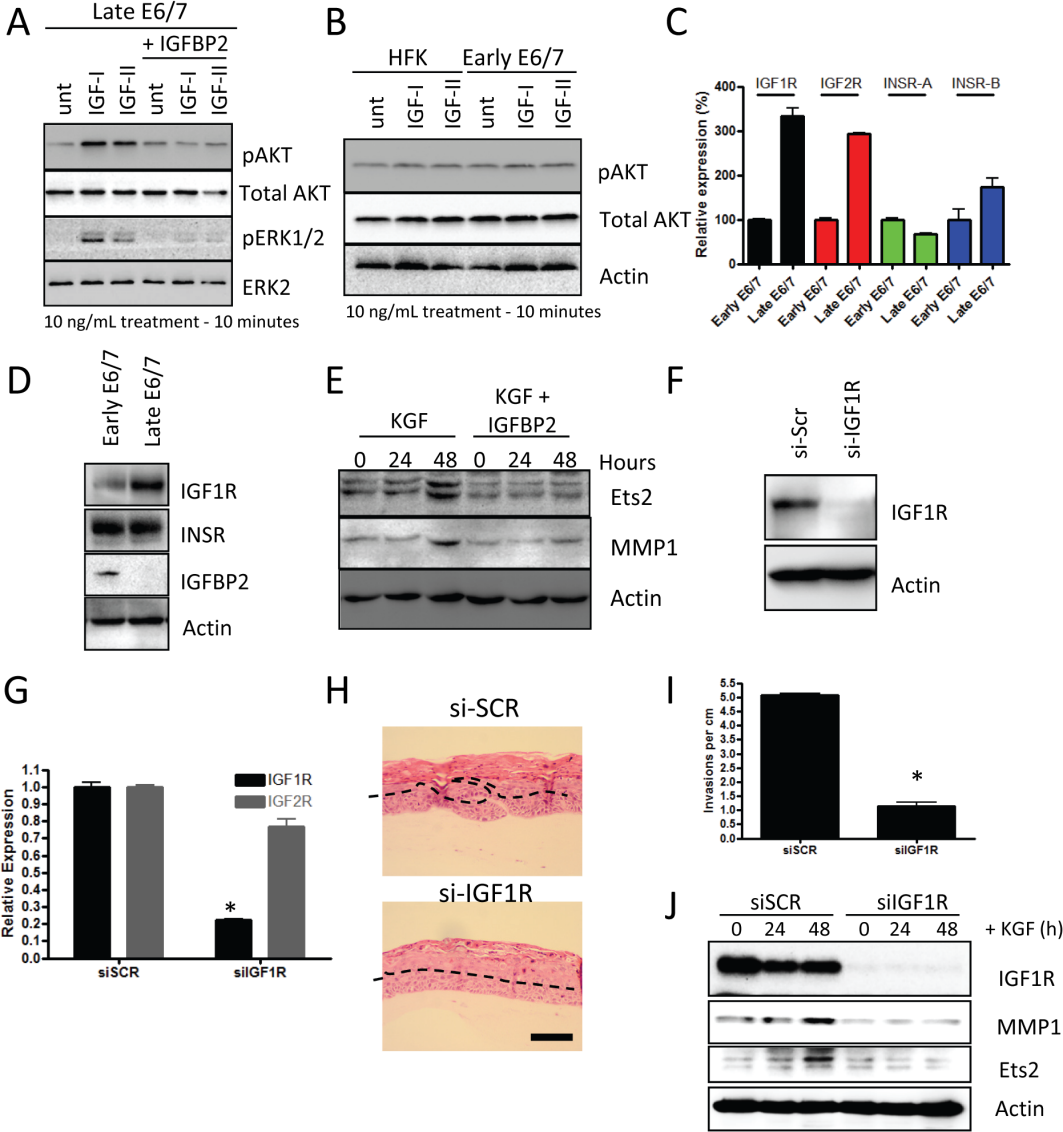
IGFBP2 blocks pro-invasive IGF1R signalling. A) Late-passage E6/7-HFKs were pre-treated with 10ng/mL IGFBP2 for 1 hour prior to 10 minutes of 10ng/ml of IGF1/2 treatment, as described in the Methods section. IGF1/2 induced activation of AKT and ERK pathways, which was inhibited by IGFBP2. B) Similar treatment of primary HFKs and early-passage E6/7-HFKs showed that these cells do not activate AKT in response to IGF1/2 treatment. C) The expression of the IGF and insulin receptors (IGF1R/2R and INSR-A/B, respectively) were assessed by real-time PCR and demonstrated significant increases in IGF1R and IGF2R mRNA. n = 3, error bars represent SEM. This was also detected at a protein level for IGF1R in cycling cultures of late passage E6/7-HFKs D). E) IGFBP2 blocks signalling pathways mediated by KGF, including those that result in activation of Ets1 and MMP1. F) The role of IGF1R signalling in regulating invasion was assessed by depletion of IGF1R using siRNA (siIGF1R) and knockdown confirmed by western blotting (F) and real-time PCR (G). n = 3, error bars represent SEM. H) siIGF1R treatment inhibited the invasion process, as quantified in I). Average of three experiments, error bars represent SEM. J) IGF1R knockdown inhibited the KGF-dependent activation of Ets2 and MMP1. Scale bars represent 100 μM.

Having established that IGFBP2 expression is lost in late passage E6/7-HFKs we next wanted to identify the significance of this loss and how it affected downstream signalling in the invasive cells. Since IGFBP2 has been shown to function through preventing IGF1 and IGF2 from binding to the IGF1-receptor [36–38], we treated E6/7-HFKs with IGFBP2 prior to IGF1/2 treatment and established that IGFBP2 is acting to block IGF1/2 induced AKT and ERK activation (Fig 6A). However, primary human foreskin keratinocytes (HFK) and early-passage E6/7-HFKs were unresponsive to IGF1 and IGF2 treatment (Fig 6B). These results implied that IGF-signalling is enhanced in the invasive cells, as a result of the loss of IGFBP2. The expression of the IGF-receptors 1 and 2 (IGF1R and IGF2R) in invasive versus non-invasive E6/7-HFKs was assessed by real-time and Western blot analysis and shown to be elevated in invasive cells whereas the related insulin receptor was unaltered (Fig 6C and 6D). This also mirrors observations that IGF1R expression is elevated in CIN3 cervical lesions [13,39].

Previous work has demonstrated that invasion of the E6/7-HFKs relies on the secretion of keratinocyte growth factor (KGF or FGF7) from stromal fibroblasts acting on the Fibroblast growth factor receptor 2b (KGFR/FGFR2b) on epithelial cells [22]. Therefore, we next investigated whether IGFBP2 could alter these effects. We treated late-passage E6/7-HFKs with KGF in the presence or absence of IGFBP2 and showed that IGFBP2 was sufficient to inhibit KGF induction of ETS2 and MMP1, known modulators of the invasive process (Fig 6E) [40,41]. This implied a crucial role for the IGF pathway in the regulation of invasion. To test this hypothesis, IGF1R levels were depleted by siRNA in invasive late-passage E6/7-HFKs and depletion confirmed at the protein and mRNA levels (Fig 6F and 6G). Depletion of IGF1R in the epithelium significantly reduced the frequency of invasions in organotypic rafts, suggesting that the IGF signalling pathway is pro-invasive (Fig 6H and 6I). We also tested whether IGF1R-depleted cells respond to the KGF pro-invasive stimulus, and similar to IGFBP2 treatment of these cells, KGF was unable to activate ETS2 and MMP1 in the absence of IGFR1 (Fig 6J).

**Fig 7:**
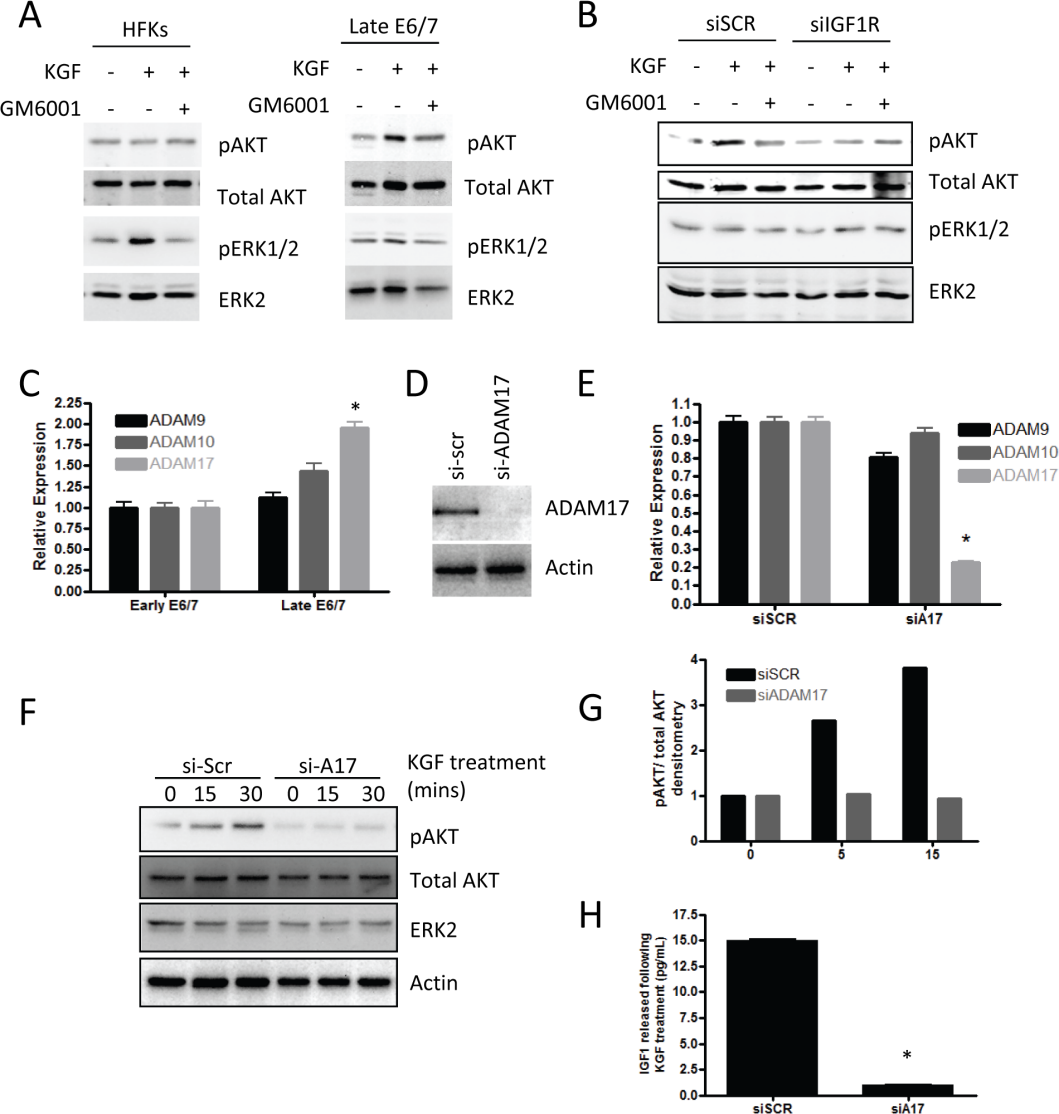
Signalling mediated by KGF requires IGF1R. A) KGF treatment of primary HFKs leads to activation of the AKT pathway in late-passage E6/7-HFKs only. B) siRNA depletion of IGF1R (siIGF1R) prevents the activation of AKT by KGF suggesting that IGF1R forms part of the signalling pathway. C) ADAM17 expression is elevated in late-passage, invasive, E6/7-HFKs, compare to early-passage E6/7-HFKs. n = 3, error bars represent SEM. D) Knockdown of ADAM17. n = 4, representative experiment shown, error bars represent SD, confirmed by western blot and real-time PCR (E) resulted in inhibition of AKT activation by KGF (F), as quantified in (G). H) Following 30 mins treatment of KGF, IGF1 was shed from late-passage E6/7-HFKs in an ADAM17 dependent manner. n = 4, error bars represent SEM.

The ability of IGF signalling to alter the pro-invasive signalling of KGF implied that the two pathways were connected and it has recently been reported that KGF functions in a protease dependent manner, specifically activating A Disintegrin And Metalloprotease 17 (ADAM17) [42]. We also confirmed in our cells that KGF activates downstream signalling events in a protease dependent manner, using the protease inhibitor GM6001 (Fig 7A). Furthermore, we found that by depleting IGF1R in the late-passage E6/7-HFKs the activation of AKT was inhibited following KGF treatment, suggesting that KGF-induced activation of AKT is both a protease-dependent and IGF1R-dependent process (Fig 7B). Late-passage E6/7-HFKs were compared to early-passage cells in terms of their expression of the ADAM family of proteins and ADAM17 expression was found to be elevated in these cells (Fig 7C). We then tested whether KGF induced activation of AKT is ADAM17 dependent using siRNA. ADAM17 was efficiently depleted (Fig 7D and 7E) and this prevented activation of the AKT pathway (Fig 7F and 7G). ADAM17 has been shown to shed various growth factors from cells, including IGF [43] so we determined if IGF is secreted from E6/7-HFKs following KGF treatment. IGF was found to be secreted from invasive E6/7-HFKs on KGF treatment, however, following knockdown of ADAM17 this secretion was inhibited (Fig 7H), implying that KGF induced ADAM17 activation leads to enhanced shedding of IGF from invasive cells and drives activation of the AKT pathway through activation of IGF1R.

**Fig 8:**
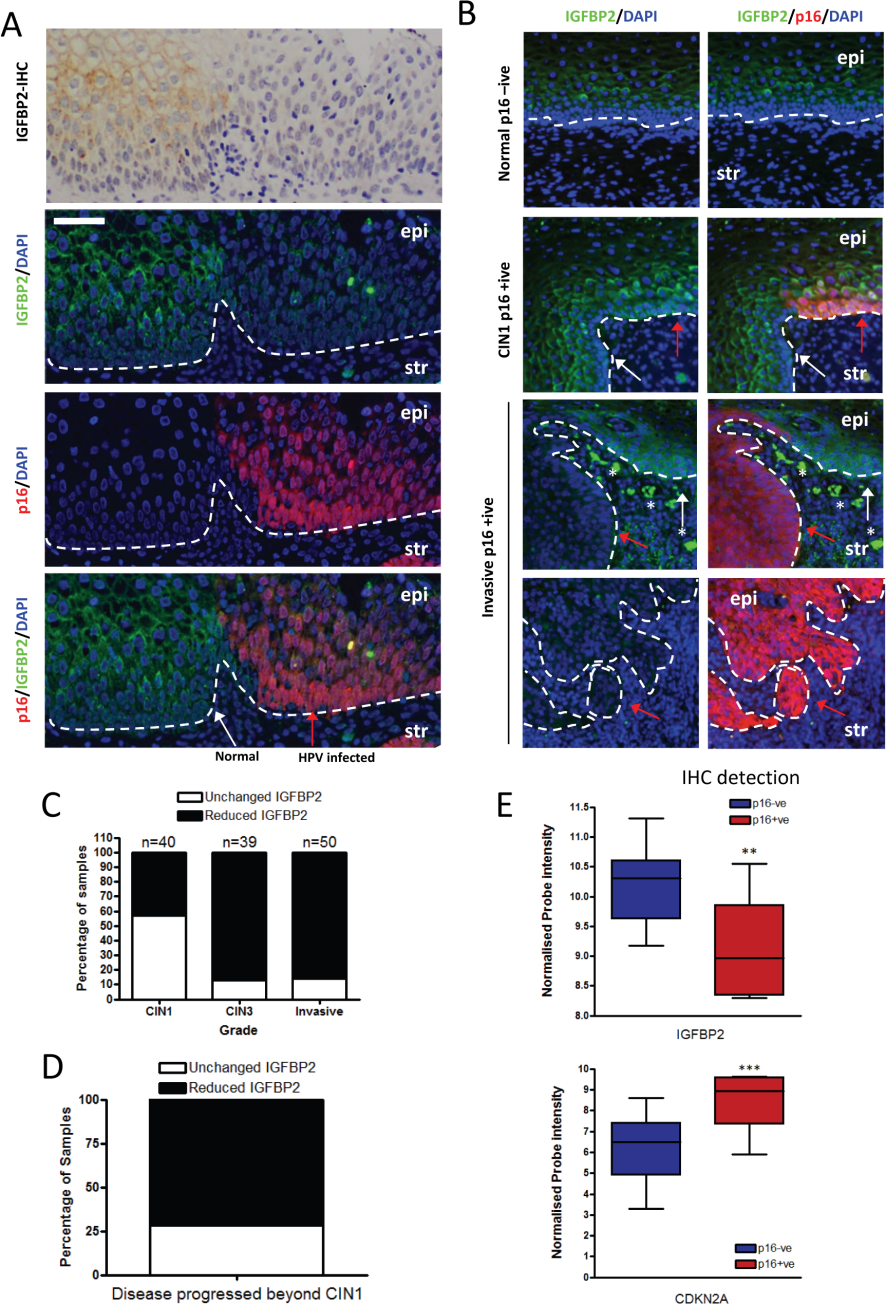
IGFBP2 is frequently down regulated in HPV16 infected CIN3 lesions. A) Immunohistochemical (IHC) and immunofluorescence staining of IGFBP2 localises the protein in the cytoplasm of epithelial cells of the cervix with regions of low IGFBP2 observed. In order to assess whether these were in HPV infected regions the same sample was co-stained with IGFBP2 and p16, the latter a surrogate marker of HPV infection. IGFBP2 was found to be commonly down-regulated in HPV infected regions (CIN3). HPV infected epithelium is indicated with a red arrow, and neighbouring normal tissue with a white arrow. Epi = epithelium; str = stroma. B) To further address the role of IGFBP2 in the progression of cervical cancer, 40 CIN1, 39 CIN3 lesions and 50 invasive carcinomas were assessed for IGFBP2 staining intensity and scored as-, +, ++, +++, for normal and p16 positive regions. If IGFBP2 scores decreased by a factor of 2 or more between p16 positive compared to normal regions then samples were identified as ‘reduced IGFBP2’. Shown are representative images of CIN1 where IGFBP2 expression was un-altered and invasive disease where IGFBP2 is reduced in p16 positive regions compared to neighbouring normal epithelium *indicates non-specific staining of red blood cells. Arrows are as stated above. C) Quantification across all samples. D) We had data for 13 patients who had low levels of IGFBP2 in the original CIN1 biopsy. 10 patients progressed, whilst 1 patient regressed and 2 had persistent CIN1 lesions. E) Analysis of publically available microarray datasets for IGFBP2 in oro-pharyngeal cancers demonstrated that in tumours where p16 is readily detected by IHC, IGFBP2 is reduced compared to tumours with no p16 staining. p16 expression in these dataset was also assessed by mRNA expression and was used as a surrogate of HPV infection.

In order to establish the clinical relevance of our findings, expression of IGFBP2 was assessed in pre-malignant cervical intraepithelial neoplasia’s (CIN) by immunohistochemistry and dual immunofluorescence, utilising p16^INK4A^ (p16) staining to distinguish premalignant cells from normal cells. IGFBP2 was readily detected in uninfected normal cervical epithelium (Fig 8A, p16 negative region, white arrow), however in regions where HPV16 had infected the epithelium, IGFBP2 was dramatically reduced in CIN3 lesions (Fig 8A, p16 positive regions, red arrow). Following our initial observations we evaluated IGFBP2 expression in a blinded manner in 40 CIN1 lesions and 39 CIN3 lesions, all of which had previously been identified as HPV16 positive [44]. IGFBP2 was found to be down-regulated in 43% of CIN1 lesions whilst in CIN3 lesions, 85% of the samples showed down-regulation (Fig 8B and 8C). There was a significant difference in the expected ratio of samples with IGFBP2 loss when comparing CIN1 and CIN3 lesions using the Fischer’s exact test, Chi-square test and Z-test (p<0.001). IGFBP2 was also found to be down-regulated in invasive disease (Fig 8B and 8C), although there was no difference in the proportions of tumours with reduced IGFBP2 at the various stages examined (Fig 8D). We had follow-up data for 13 patients with CIN1 where IGFBP2 was reduced. From this group, 10 patients progressed to CIN3, the other 3 patients either regressed (1 case) or remained CIN1 (2 cases) (Fig 8E). The results imply that IGFBP2 is commonly down-regulated at advanced stages of infection, correlating with the effects of prolonged HPV16 E6 and E7 expression and reduced levels of IGFBP2 in CIN1 disease may indicate a propensity to progress to a high grade. We have also investigated whether IGFBP2 expression is regulated in other cancers associated with HPV infection. HPV infection has been observed in head and neck cancers, where it is associated with between 30–60% of oro-pharyngeal cancers [45]. We have utilised publically available gene array datasets to assess the expression of IGFBP2 in oro-pharyngeal cancers [46]. In these studies HPV infection was detected by immunohistochemistry of the surrogate marker p16 and the samples were sub-divided into p16 positive and p16 negative groups and microarray datasets were analysed for IGFBP2 and p16 expression. In p16 positive cancers, IGFBP2 expression was significantly reduced (Fig 8F), suggesting that down-regulation of IGFBP2 expression is likely associated with HPV infection in oro-pharyngeal cancers.

**Fig 9:**
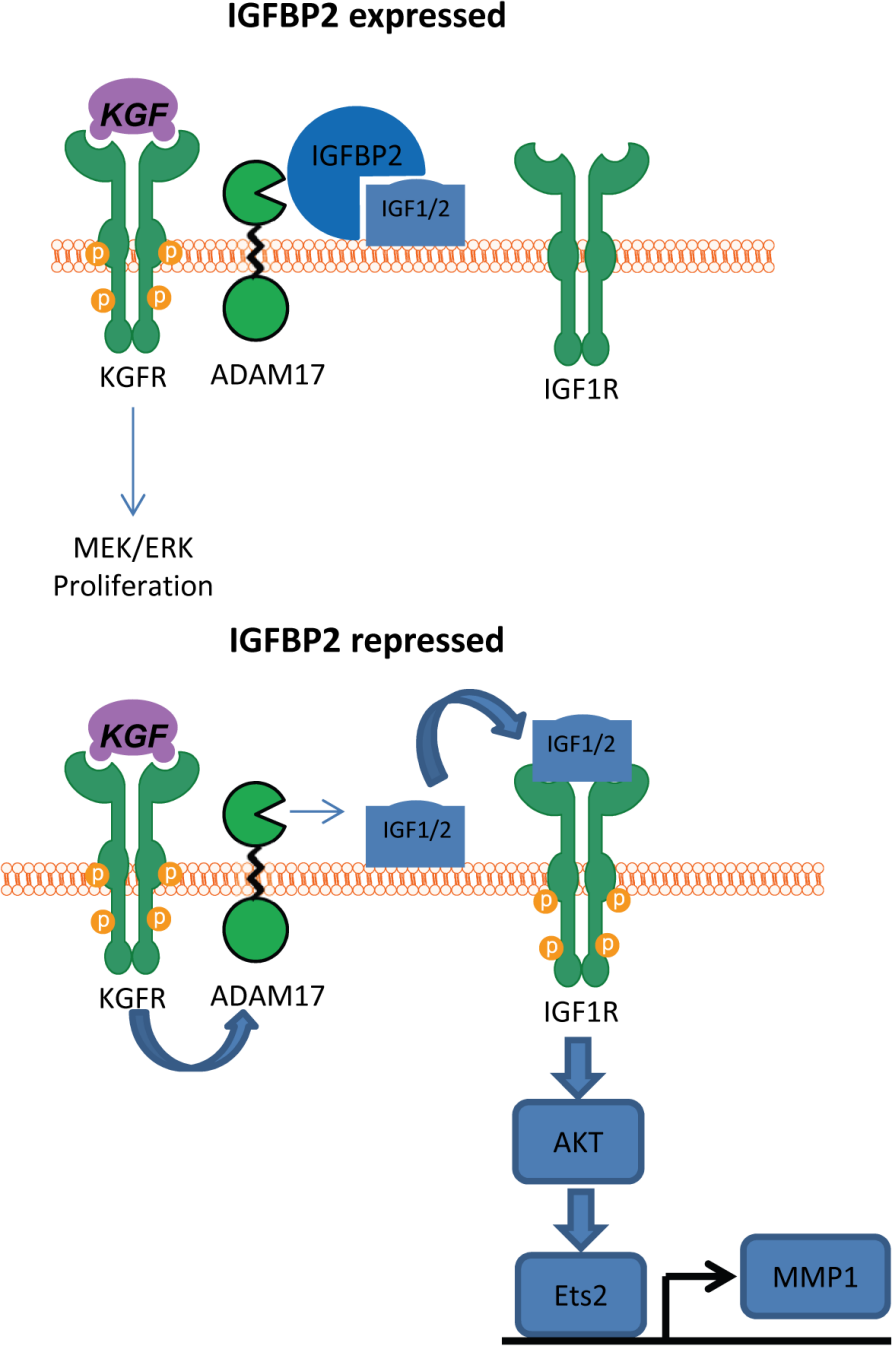
Proposed mechanism of IGFBP2 function. In the presence of IGFBP2, IGF1/2 cannot be released from the cell surface and therefore cannot activate the IGF1 receptor. However, when IGFBP2 is lost, KGF activation of ADAM17 leads to cleavage of the unprotected IGF1 leading to IGF1R activation and subsequent AKT activation, which we have previously demonstrated leads to expression of MMP1 [22].

In summary, we have shown that IGFBP2 is reduced in an E6/7 dependent manner over the passage of human keratinocytes leading to invasion in our 3-dimensional model system. Our *in vivo* studies with cervical premalignancies show that IGFBP2 expression is reduced with severity of disease from CIN1 to CIN3. The reduced IGFBP2 expression leads to activation of the IGF/IGFR pathways, which through cross talk with the KGF/FGFR2b complex can drive invasion (Fig 9). The results suggest that the IGF/IGFR/IGFBP2 axis would make a logical target for further investigation for potential treatment of cervical cancers.

## Discussion

Invasion of the hyper-proliferative cervical epithelium into the surrounding stroma is an important event in progressive disease and here we have identified a crucial role for IGFBP2 in controlling this invasion process. Our results suggest that the prolonged expression of E6/7 proteins can generate an invasive epithelium through depletion of IGFBP2 expression, which in turn leads to signalling through the IGF1R in cross-talk with the FGFR2b. These results are also in keeping with previous results which demonstrated that HPV16 E7 can transform fibroblasts, in an IGF1R dependent manner [47]. We propose that IGFBP2 functions as a brake preventing the HPV-infected epithelium from invading into the underlying stroma.

Our *in vitro* findings are mirrored in cervical cancer specimens where IGFBP2 expression is commonly lost in 85% of CIN3 lesions, which progress to invasive disease with high incidence, if left untreated [48], while CIN1 lesions do not. We did however observed that 43% of CIN1 lesions have reduced IGFBP2, and our preliminary data indicates that a significant proportion of patients with CIN1 disease who later progressed to a higher grade lesion, had reduced levels of IGFBP2 in the HPV infected epithelium of the original CIN1 biopsy. As this has only been examined in a limited number of cases, future studies are required to confirm that IGFBP2 levels may indicate patients at risk of progression. If the reduction of IGFBP2 levels identifies a sub-group of CIN1 lesions that have the propensity to progress, this could be useful clinically, as these patients could be monitored more closely to detect disease progression. There is a possibility that the CIN1 lesions where IGFBP2 were down regulated were mis-classified, however, grading of lesions was conducted by two pathologists with overall agreement in each case.

Mechanistically our results demonstrate a critical role for IGF-signalling in driving the invasive process. The IGF pathway is well known to be modulated in cancer and is known to promote neoplastic growth [13,39]. The IGF receptors are expressed in a variety of cancers, and *in vivo* studies have demonstrated that cancer cells have a dependency for IGF1. Following prolonged expression of HPV16 E6 and E7 the IGF pathway becomes activated i) through loss of IGFBP2 and ii) through enhanced expression of the IGF-receptors. Here we demonstrate that re-addition or re-expression of IGFBP2 through HDAC inhibitor treatment, blocks IGF-signalling and is sufficient to inhibit epithelial invasion, while reciprocal knockdown of IGFBP2 in non-invasive E6/7-HFKs resulted in enhanced invasion, demonstrating a critical role for the pathway in the invasion process. We have further demonstrated that the loss of IGFBP2 allows pro-invasive signals derived from the stroma to enhance epithelial invasion, and this is conducted via IGF1R. It has previously been demonstrated that the keratinocyte growth factor functions via activating the metalloprotease ADAM17 [42] and here we show this is also the case, and there is preferential activation of the AKT pathway. We have previously demonstrated that the AKT pathway is activated in cervical cancer specimens [6] and have demonstrated it as a key component of epithelial invasion [22]. Here we show that the activation of AKT by KGF was dependent on IGF1R (Fig 9), and this can be modulated by IGFBP2 and ADAM17. Signalling via the IGF1R pathway has been proposed to be an important determinant of cervical cancer progression, since elevated expression of IGF1R has been observed in cervical specimens which positively correlated with stage of the CIN lesions [13,39] and further highlights the importance of the IGF-axis in HPV infection and incidence of CIN lesions [49]. This, together with our data, suggests that an activated IGF1R pathway promotes a pro-invasive phenotype in the cervical epithelium. An important caveat is that our *in vitro* model utilises fibroblasts which promote epithelium invasion [22], and reduction of IGFBP2 in the epithelium alone, may not be sufficient to drive invasion *in situ*. As CIN lesions take a number of years to progress to invasive disease during this time the stroma may be ‘activated’ which in combination with loss of IGFBP2 can drive epithelium invasion. In line with this hypothesis detection of cancer associated myofibroblasts has been observed in the stroma of cervical cancers and is correlated with poor prognosis [50].

IGFBP2 has been demonstrated to have both tumour suppressive and oncogenic properties in different cancer types. Our data show IGFBP2 as an inhibitor of the invasion process in our 3-D model of cervical pre-cancer and in the main, IGFBP2 tumour suppressive functions are those which antagonise IGF signalling [38,51,52], although IGF-independent pro-apoptotic functions of IGFBP2 have also been described [53]. In examples where IGFBP2 functions in an oncogenic manner, these functions appear to be independent of IGF and are mediated via integrin alpha 5 [54,55] which leads to inhibition of PTEN and ultimately activates the AKT pathway [56]. These oncogenic functions of IGFBP2 were not observed in our studies which may be due to functions of the HPV E6 and E7, which have been shown to down-regulate various integrins, including alpha 5 [57]. Whilst the HPV vaccine will ultimately reduce the incidence of cervical cancer if administered universally, there still remains a generation of women who will require intervention. Since IGFBP2 is itself a potential target for therapeutic intervention [58], and has been shown to inhibit the growth of breast cancer cells *in vivo* [59], we propose that since HDAC3 is involved in reducing expression of IGFBP2, HDAC inhibitors maybe a useful tool to treat patients with progressive disease.

## Methods

### Ethics statement

Ethical approval for human materials used in this study was from the Northern Ireland Tumour Bank (NIB11-0001).

### Cell culture

Primary human foreskin keratinocytes, derived from neonatal foreskins were maintained in Epilife containing growth supplement (Life-Technologies). Primary human foreskin fibroblasts (Cascade Biologics), the cervical cancer cell lines: C33a and Caski (from existing laboratory stocks, frozen at low passage), and Hela’s (American Type Culture Collection) were maintained in DMEM with 10% FBS. hTERT immortalised HFKs were obtained from the Rheinwald lab and maintained in E-medium with mitomycin C arrested J2-3T3 feeders. Immortalisation of HFKs using J2-3T3 and Rock inhibitor, Y27632, was conducted as previously described [23] (n = 2), to compare IGFBP2 expression levels all cells were grown in E-medium with J2-3T3s.

### Reagents

Recombinant IGFBP2, IGFBP3, IGF-I, IGF-II, KGF (Peprotech) and protease inhibitor, GM6001 (Millipore) were prepared according to manufacturer’s protocols. Short term treatments were conducted in confluent monolayers generated following 2 days growth of 200,000 HFKs in 6 well plates and medium was replaced with Epilife without growth supplement for 24 hours before treatment with individual growth factors. To assess the effects of IGFBP2 on IGF1/2 signalling, HFKs were grown as above then switched to low glucose, serum free DMEM for 24 hours and samples pre-treated for 1 hour with IGFBP2 prior to IGF1/2 treatment. Samples were lysed in RIPA buffer for 20 minutes on ice. To assess the impact of KGF on ETS2 and MMP1 induction confluent monolayers grown on 100 μg/mL collagen I before commencing KGF treatment in fresh Epilife with growth supplement. Proteins were harvested using urea buffer. Organotypic cultures were grown in the presence of pRb-depleted fibroblasts in E-medium as previously described [5], stained with haemotoxylin and eosin and invasions per cm of raft were counted. In Fig 1B and 1H media was changed each day. A modified organotypic system was used for the Caski cell line as described previously [60] with the exception that matrigel was replaced with an equivalent volume of egg-white, as previously described [61]. Real-time PCR was conducted using Roche Lightcycler480, primers are described in S1 Table and gene expression data was normalised to RPLPO.

### Generation of primary cell lines and siRNA

E6/7-HFKs and pRb-depleted HFFs were generated as previously described [6]^,^ [22,62]. shIGFBP2#1 and #2 were from the pRSC retrovirus shRNA library (UCL) with the following target sequences, GTGGAGAACCACGTGGACA and CGGAGCAGGTTGCAGACAA. siRNA sequences used in this study were; siE6/7: GCACACACGUAGACAUUCGdTdT as previously described [63]. siHDAC1 and 2 were from Qiagen. siHDAC3: GAUGCUGAACCAUGCACCUTT as previously described [64]. siIGF1R: CAAUGAGUACAACUACCGCdTdT as previously described [65], siRNA targeting ADAM17 was purchased from Dharmacon siADAM17 smartpool. siRNA transfections were conducted using RNAiMAX (Life-Technologies).

### Antibodies, arrays, ELISA and CHIPs

Immunofluorescence (IF) and immunohistochemistry staining procedures were previously described [22]. Cervical intraepithelial neoplasia samples (CIN1 and CIN3) were from New Mexico and invasive cervical cancers were from Belfast Health and Social trust and a TMA containing tumour and matched normal tissue (Abcam, Ab178142). IGFBP2 staining intensity and scored as-, +, ++, +++, for normal and p16 positive regions. If IGFBP2 scores decreased by a factor of 2 or more between p16 positive compared to normal regions then samples were identified as reduced IGFBP2. Antibodies used for IF were as follows: IGFBP2 (Cell-Signaling Technologies #3922, 1:200), p16 (BD-Biosciences, 554079, 1:200). Western blot analysis utilised the following antibodies: Cell-Signaling Technologies: IGFBP2 (#3922, 1:1000), phospho-AKTser473 (#4060, 1:2000), total AKT (#2920, 1:1000) HDAC1 (#5356, 1:1000) and HDAC2 (#2545, 1:1000), Santa-Cruz: p53 (sc-126, 1:1000), pERK1/2 (sc-7383, 1:1000), ERK2 (sc-154-G, 1:1000), IGF1R (sc-713, 1:1000), INS-R (sc711, 1:1000), Ets2 (sc-351, 1:2000), MMP1 (sc-58377, 1:1000) and HDAC3 (sc-11417, 1:1000), Sigma-aldrich: beta-actin (A2228, 1:10,000) and ADAM17 (SAB3500367, 1:1000) NcoR1 (Bethyl laboratories A301-145A, 1:1000) and NcoR2 (Abcam Ab24551, 1:1000). Human Growth factor array from RayBiotech was conducted according to manufacturer’s protocols and arrays were analysed using densitometry software, Fluorchem SP. Human IGF-1 ELISA was from RayBiotech. CHIP-qPCR experiments were conducted as previously described [66] using 3 x10^6^ cells per immunoprecipitation. Chip antibodies used in this study as stated above, HDAC3 (2μg per CHIP), NcoR1 (2μg per CHIP), NcoR2 (10μL per CHIP) and H3K9Ac (2.5μg per CHIP). Real-time PCR for CHIP-qPCR was conducted using Roche Lightcycler480, primers are described in S2 Table.

## Supporting Information

S1 Fig Basement membrane is degraded in invasive regions of organotypic rafts. Organotypic raft cultures of early and late passage E6/7-HFKs, stained with the basement membrane markers Collagen VII and Laminin 5. Both cell types produce a basement membrane during 14 day cultures (white arrows) however these proteins are not detected at the interface of the epithelium and stroma in invasive regions of late passage E6/7-HFK cultures (Red arrows).
(PDF)

S2 Fig HPV16 E6/7 regulates IGFBP2 in primary and immortalised HFKs. A) Two additional independently generated E6/7 lines were generated and Western blots conducted to assess IGFBP2 expression levels. B) Control HFKs (pBabe) and E6/7-HFKs were generated and passaged in either Epilife or in co-culture with J2-3T3 fibroblasts in E-medium, protein lysates at the indicated passage were analysed for IGFBP2 expression by Western blot. C and D) Western blot and real time PCR analysis of IGFBP2 expression in normal HFK (passage 4), hTERT immortalised keratinocytes, keratinocytes immortalised by co-culture with Y27632 (passage 18) and late passage E6/7-HFKs (passage 20) E and F) Western blot and real-time PCR analysis of IGFBP2 protein and mRNA levels following E6/7 expression in immortalised keratinocytes. Lysates and RNA were prepared from cells harvested four passages after selection for transduced cells.
(PDF)

S3 Fig Depletion of E6/7 restores IGFBP2 expression. A) Both HPV16 E6 and E7 mRNA are expressed in early and late passage E6/7-HFKs as detected by real-time PCR. We consistently observed increased expression of E6 and E7 in late passage cells. B) Western blot analysis following depletion of E6/7 from late passage HFKs. siRNA targeting E6 and E7 resulted in inhibition of E6/7 functions as observed by an increase in expression of p53 and Rb and an enhanced expression of IGFBP2. There is additional regulation of IGFBP2 expression by glucose as previously reported [1]. E-medium is a high glucose medium used to grow organotypic cultures as described in the Materials and Methods section. C) H+E staining of sectioned organotypic raft cultures of late passage E6/7-HFKs treated with scrambled siRNA (siSCR) or siRNA targeting HPV16 E6 and E7. siE6/7 leads to suppression of invasion as quantified in (D). Scale bars represent 100 μM.
(PDF)

S4 Fig IGFBP2 suppresses invasion in organotypic raft culture in late passage HFKs. A) Real-time PCR of IGFBP1 in HFFs, HFKs and early and late passage E6/7-HFKs. B) Quantification of IGFBP2 in 15 μL conditioned medium from the indicated cells, levels of IGFBP2 were compared to recombinant IGFBP2, a representative blot is shown. C) Range of IGFBP2 concentrations in medium conditioned by the indicated cells. D) Addition of IGFBP3 to organotypic cultures had a modest inhibitory effect on epithelial invasion as quantified in E), data from three independent experiments are shown, error bars represent SEM. F) A modified organotypic culture was generated to grow the HPV16 positive CASKi cell line. These cells invaded the collagen matrix as determined by sectioning and H+E stain. Addition of IGFBP2, but not IGFBP3, throughout the culture period significantly inhibited invasion of the epithelial cells into the collagen matrix. Invasion frequency is quantified in (G) from a representative experiment, error bars represent SD across three sections of the organotypic raft. Scale bars represent 100 μM. H) Real-time PCR of IGFBP2 in early and late passage E6/7-HFKs treated with 10 ng/mL Insulin, IGFI or IGFII for 24 hours.
(PDF)

S5 Fig Hypermethylation of the IGFBP2 promoter in invasive epithelial cells. A) Reduced bisulphite sequencing from ENCODE in keratinocytes identified two potential CpG islands in the IGFBP2 promoter. B) Pyrosequencing of the identified regions identified hypermethylation of Region 1 in late passage E6/7-HFKs. C) Addition of 1μM 5-aza-cytodine did not restore IGFBP2 expression in late passage E6/7-HFKs at a protein or transcriptional level (D).
(PDF)

S6 Fig HDAC inhibition restores IGFBP2 transcription in Hela cells. Addition of either 0.5 μM TSA or 5mM sodium butyrate (SB) enhanced IGFBP2 expression to levels above those expressed in the HPV negative cervical cancer cell line, C33a.
(PDF)

S7 Fig Dose response curves to HDAC inhibition. Early and late passage E6/7-HFKs were exposed to a range of doses of TSA for 72 hours. Cell viability was assessed using Alamar Blue (Life Technologies) to assess the growth inhibitory effects of TSA.
(PDF)

S8 Fig HDAC3 inhibition restores IGFBP2 expression. Late passage E6/7-HFKs were treated with a range of doses of RGFP966 for 24 hours. A) Western blot analysis identified enhanced IGFBP2 protein expression following inhibition of HDAC3, which was mirrored by enhanced expression at the RNA level (B).
(PDF)

S1 Table Real-time primers used in this study. (PDF)

S2 Table CHIP-qPCR primers used in this study (PDF)
