# Supplementary figures and images for: HPV16 Down-Regulates the Insulin-Like Growth Factor Binding Protein 2 to Promote Epithelial Invasion in Organotypic Cultures
Source: PLoS Pathog. 2015 Jun 24;11(6):e1004988. doi: 10.1371/journal.ppat.1004988 (PMC4479471; doi:10.1371/journal.ppat.1004988)

Supplemental Figure 1

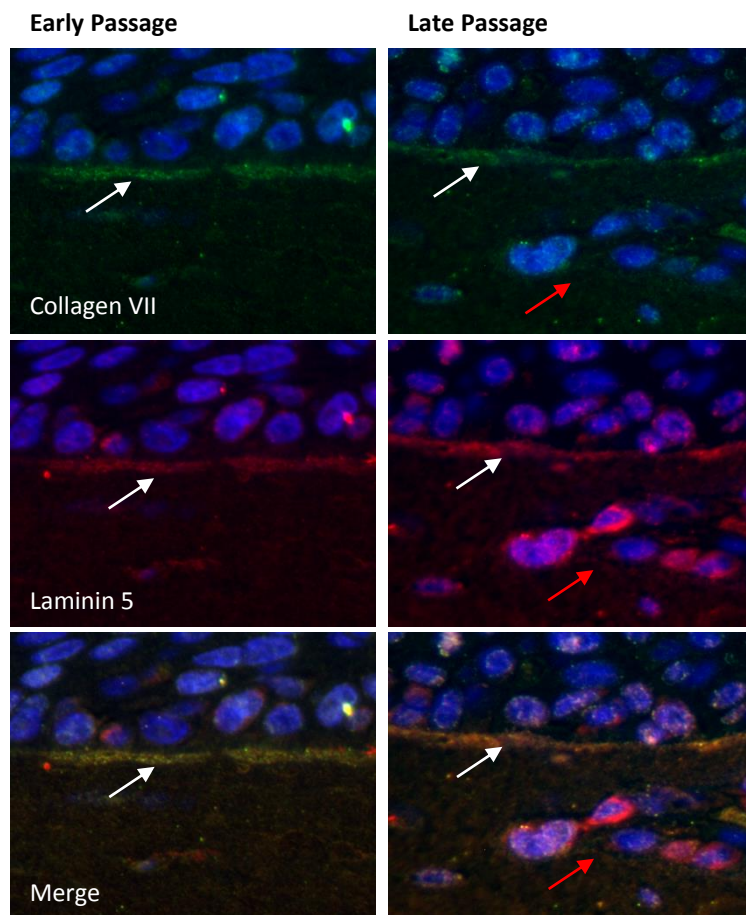

Supplement: S1 Fig — Organotypic raft cultures of early and late passage E6/7-HFKs, stained with the basement membrane markers Collagen VII and Laminin 5. Both cell types produce a basement membrane during 14 day cultures (white arrows) however these proteins are not detected at the interface of the epithelium and stroma in invasive regions of late passage E6/7-HFK cultures (Red arrows). (PDF) [file ppat.1004988.s001.pdf]

# Supplemental Figure 2

**A**

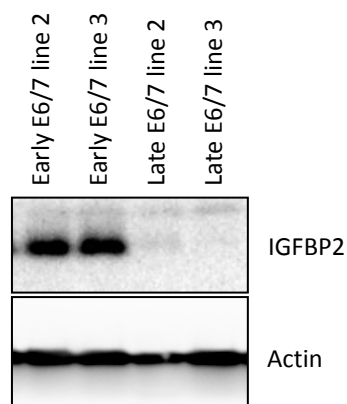

**C**

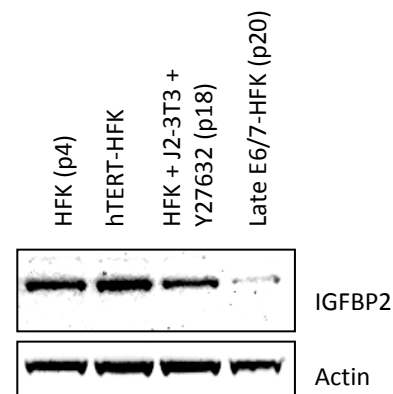

**E**

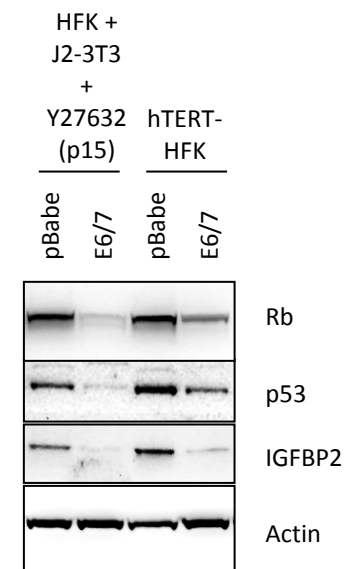

**B**

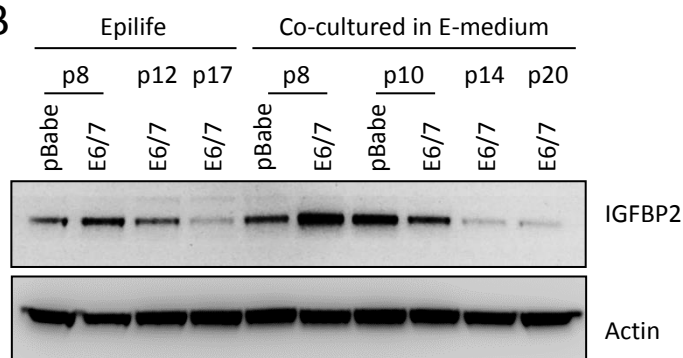

**D**

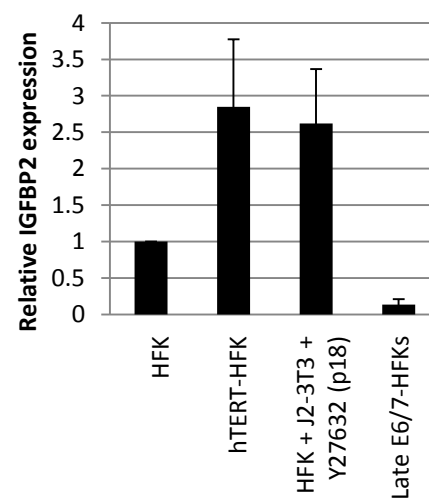

**F**

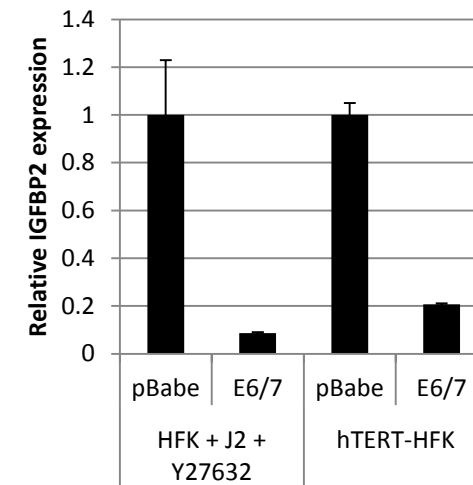

Supplement: S2 Fig — A) Two additional independently generated E6/7 lines were generated and Western blots conducted to assess IGFBP2 expression levels. B) Control HFKs (pBabe) and E6/7-HFKs were generated and passaged in either Epilife or in co-culture with J2-3T3 fibroblasts in E-medium, protein lysates at the indicated passage were analysed for IGFBP2 expression by Western blot. C and D) Western blot and real time PCR analysis of IGFBP2 expression in normal HFK (passage 4), hTERT immortalised keratinocytes, keratinocytes immortalised by co-culture with Y27632 (passage 18) and late passage E6/7-HFKs (passage 20) E and F) Western blot and real-time PCR analysis of IGFBP2 protein and mRNA levels following E6/7 expression in immortalised keratinocytes. Lysates and RNA were prepared from cells harvested four passages after selection for transduced cells. (PDF) [file ppat.1004988.s002.pdf]

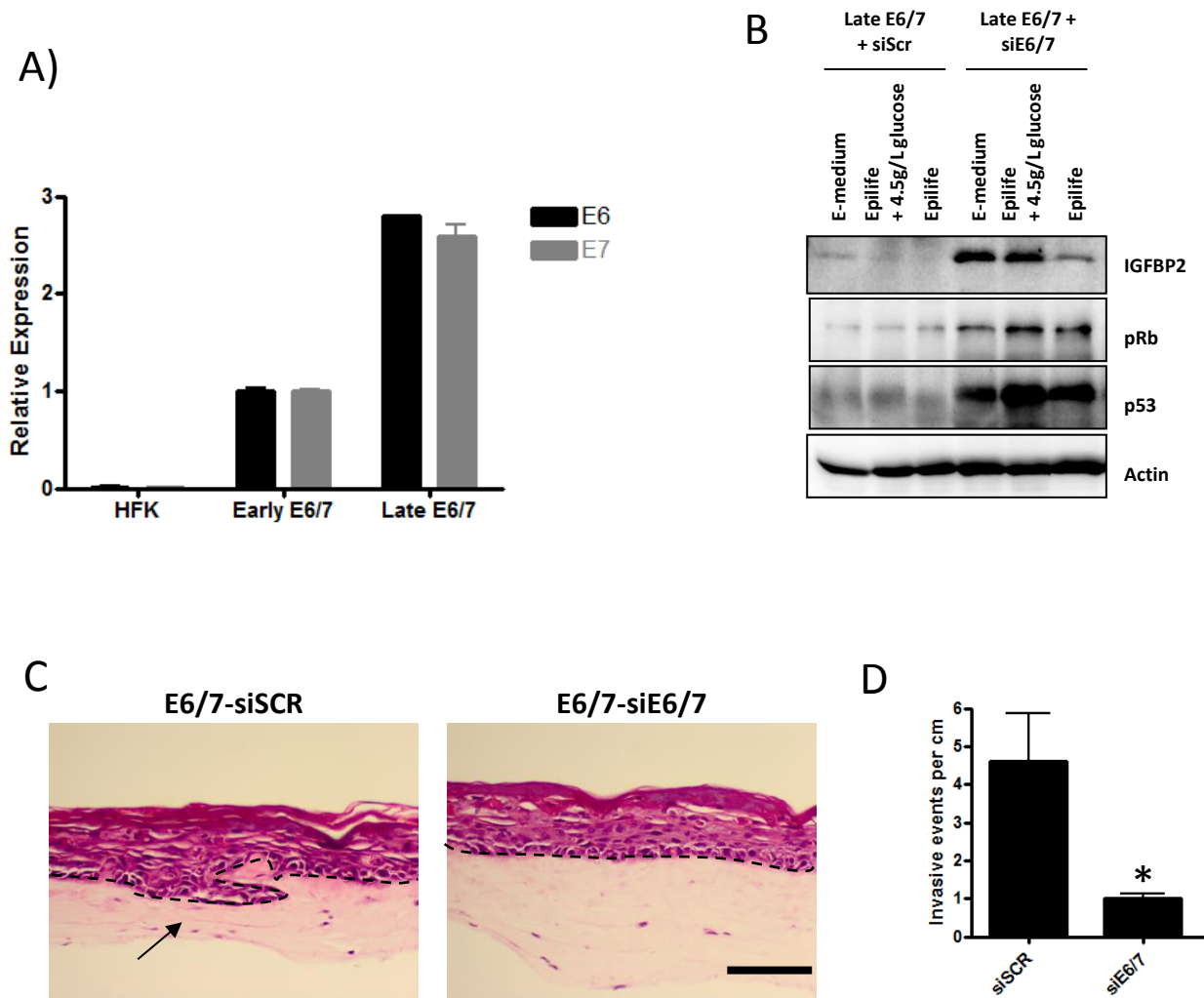

Supplement: S3 Fig — A) Both HPV16 E6 and E7 mRNA are expressed in early and late passage E6/7-HFKs as detected by real-time PCR. We consistently observed increased expression of E6 and E7 in late passage cells. B) Western blot analysis following depletion of E6/7 from late passage HFKs. siRNA targeting E6 and E7 resulted in inhibition of E6/7 functions as observed by an increase in expression of p53 and Rb and an enhanced expression of IGFBP2. There is additional regulation of IGFBP2 expression by glucose as previously reported [1]. E-medium is a high glucose medium used to grow organotypic cultures as described in the Materials and Methods section. C) H+E staining of sectioned organotypic raft cultures of late passage E6/7-HFKs treated with scrambled siRNA (siSCR) or siRNA targeting HPV16 E6 and E7. siE6/7 leads to suppression of invasion as quantified in (D). Scale bars represent 100 μM. (PDF) [file ppat.1004988.s003.pdf]

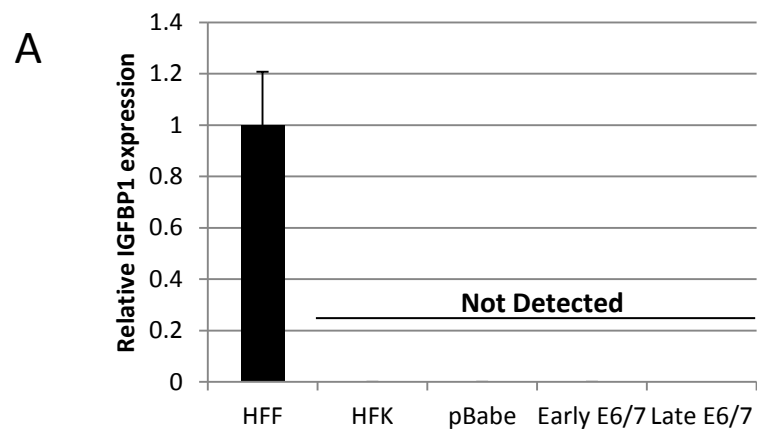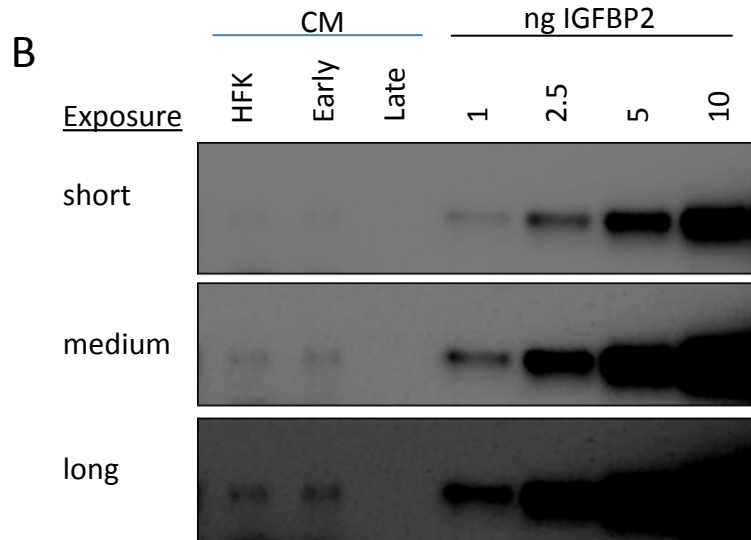

**C**

| Cell line | [IGFBP2]      |
|-----------|---------------|
| HFK       | 2.0-5.0 ng/mL |
| Early     | 3.0-5.8 ng/mL |
| Late      | 0.0-1.2 ng/mL |

**D**

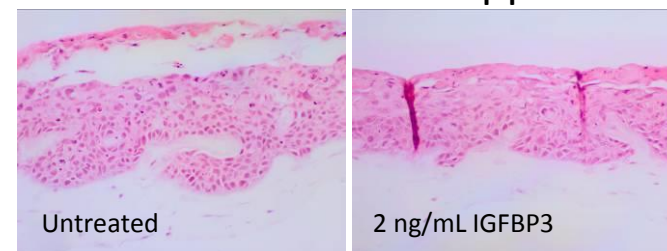

**E**

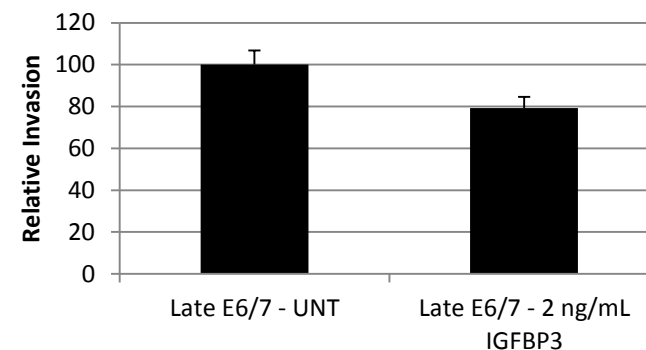

**F**

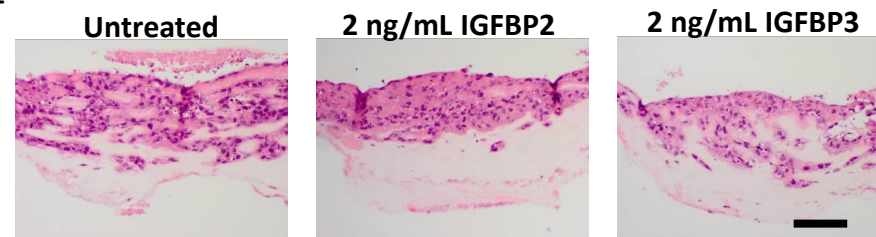

**G**

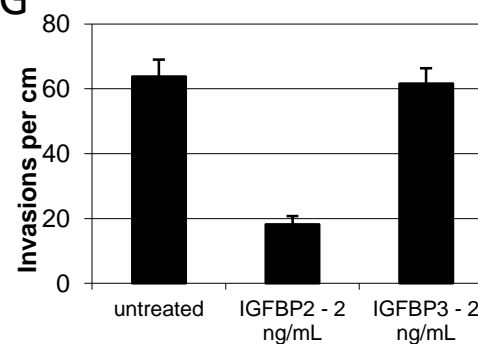

**H**

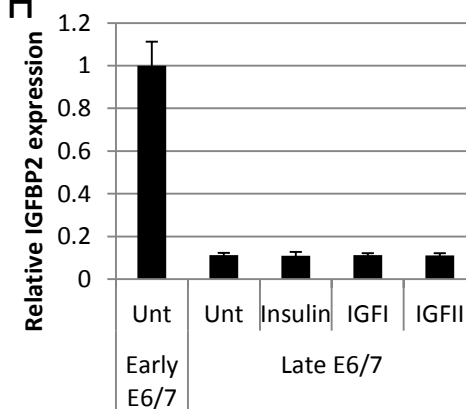

Supplement: S4 Fig — A) Real-time PCR of IGFBP1 in HFFs, HFKs and early and late passage E6/7-HFKs. B) Quantification of IGFBP2 in 15 μL conditioned medium from the indicated cells, levels of IGFBP2 were compared to recombinant IGFBP2, a representative blot is shown. C) Range of IGFBP2 concentrations in medium conditioned by the indicated cells. D) Addition of IGFBP3 to organotypic cultures had a modest inhibitory effect on epithelial invasion as quantified in E), data from three independent experiments are shown, error bars represent SEM. F) A modified organotypic culture was generated to grow the HPV16 positive CASKi cell line. These cells invaded the collagen matrix as determined by sectioning and H+E stain. Addition of IGFBP2, but not IGFBP3, throughout the culture period significantly inhibited invasion of the epithelial cells into the collagen matrix. Invasion frequency is quantified in (G) from a representative experiment, error bars represent SD across three sections of the organotypic raft. Scale bars represent 100 μM. H) Real-time PCR of IGFBP2 in early and late passage E6/7-HFKs treated with 10 ng/mL Insulin, IGFI or IGFII for 24 hours. (PDF) [file ppat.1004988.s004.pdf]

Supplemental Figure 5

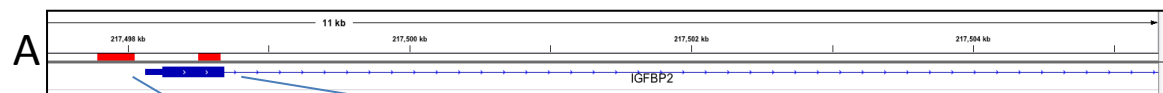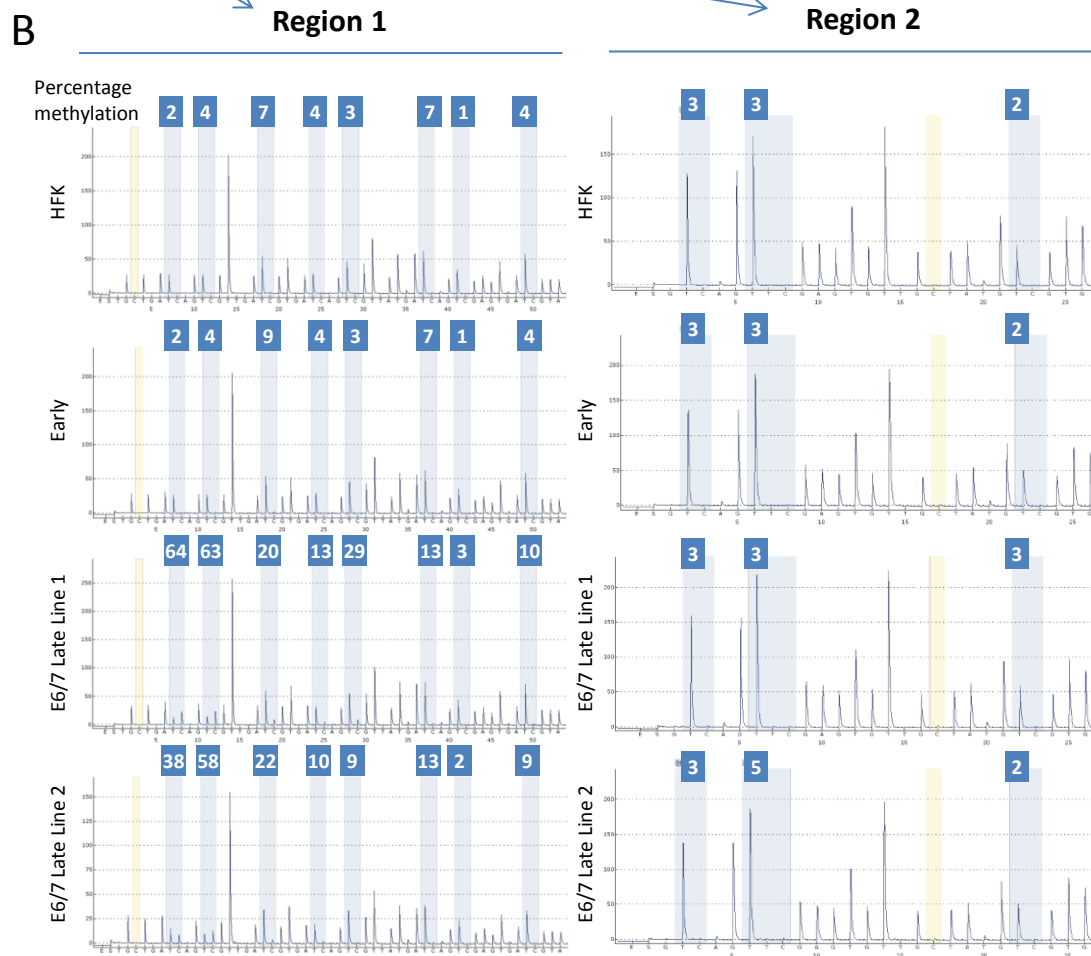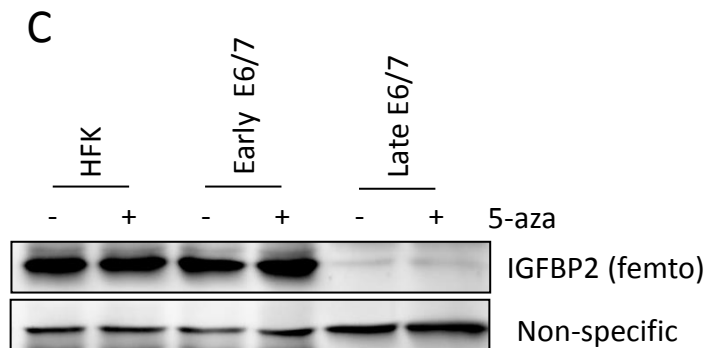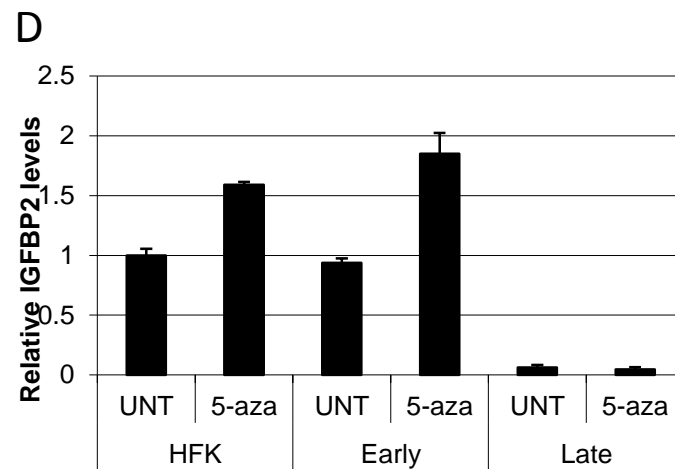

Supplement: S5 Fig — A) Reduced bisulphite sequencing from ENCODE in keratinocytes identified two potential CpG islands in the IGFBP2 promoter. B) Pyrosequencing of the identified regions identified hypermethylation of Region 1 in late passage E6/7-HFKs. C) Addition of 1μM 5-aza-cytodine did not restore IGFBP2 expression in late passage E6/7-HFKs at a protein or transcriptional level (D). (PDF) [file ppat.1004988.s005.pdf]

Supplemental Figure 6

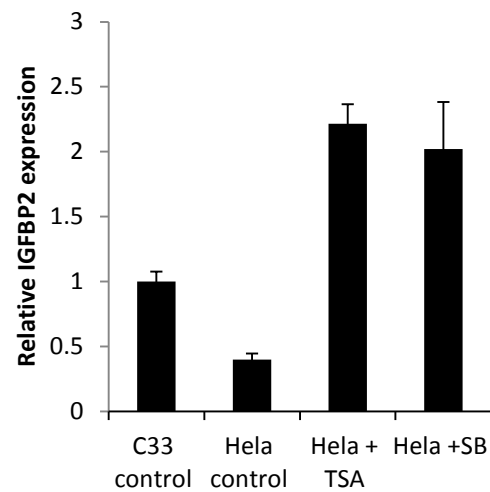

Supplement: S6 Fig — Addition of either 0.5 μM TSA or 5mM sodium butyrate (SB) enhanced IGFBP2 expression to levels above those expressed in the HPV negative cervical cancer cell line, C33a. (PDF) [file ppat.1004988.s006.pdf]

Supplemental Figure 7

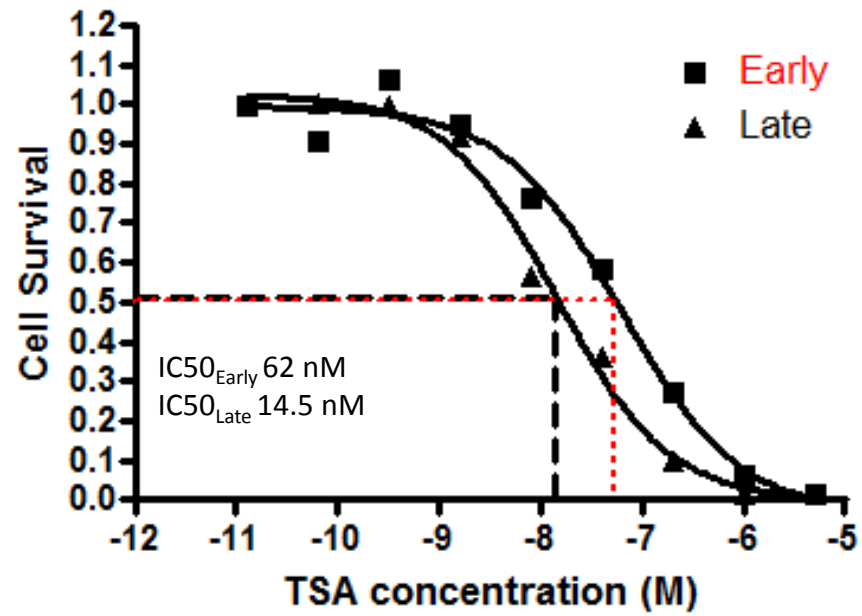

Supplement: S7 Fig — Early and late passage E6/7-HFKs were exposed to a range of doses of TSA for 72 hours. Cell viability was assessed using Alamar Blue (Life Technologies) to assess the growth inhibitory effects of TSA. (PDF) [file ppat.1004988.s007.pdf]

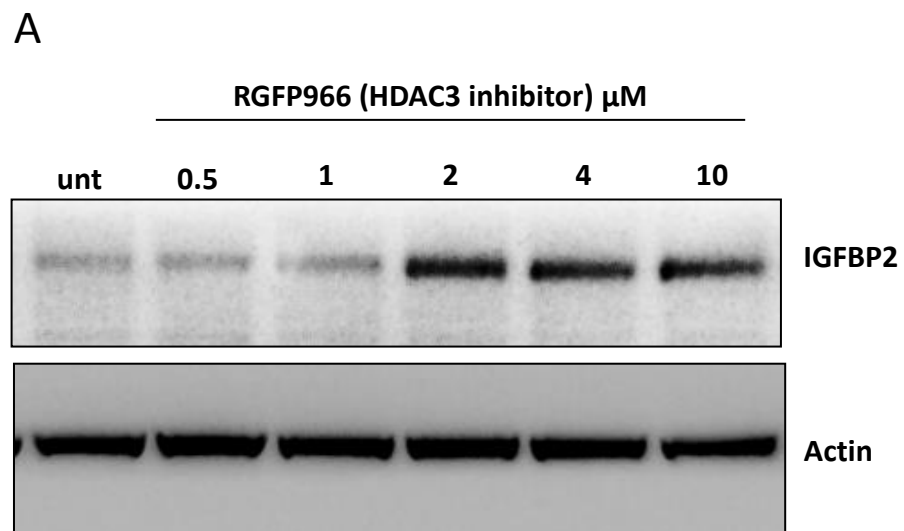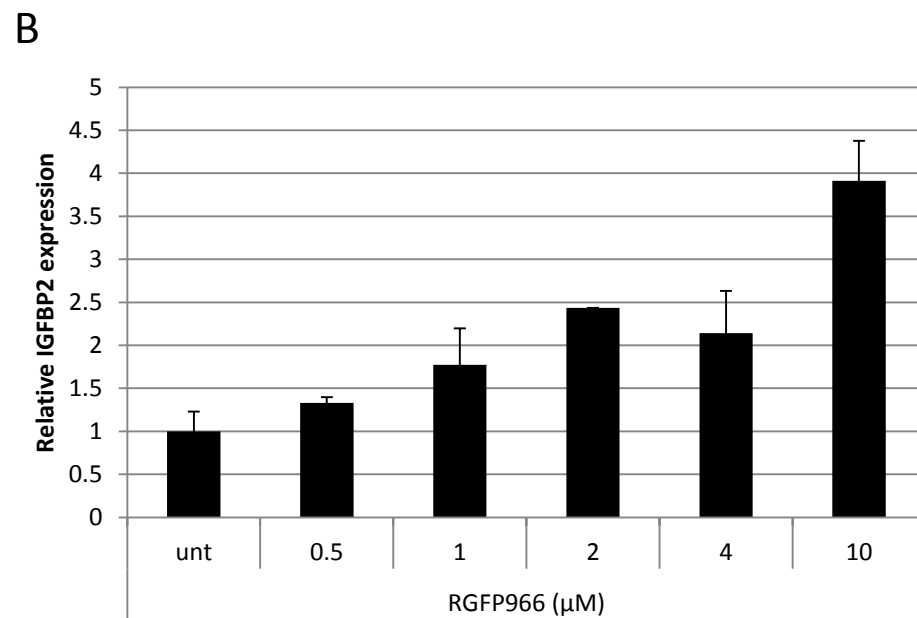

Supplement: S8 Fig — Late passage E6/7-HFKs were treated with a range of doses of RGFP966 for 24 hours. A) Western blot analysis identified enhanced IGFBP2 protein expression following inhibition of HDAC3, which was mirrored by enhanced expression at the RNA level (B). (PDF) [file ppat.1004988.s008.pdf]
