# Supplementary material for: HPV16 Down-Regulates the Insulin-Like Growth Factor Binding Protein 2 to Promote Epithelial Invasion in Organotypic Cultures
Source: PLoS Pathog. 2015 Jun 24;11(6):e1004988. doi: 10.1371/journal.ppat.1004988 (PMC4479471; doi:10.1371/journal.ppat.1004988)
Supplement: S1 Table — (PDF) [file ppat.1004988.s009.pdf]

Supplemental Table 1: Real-time primers used in this study

cDNA target	Forward primer	Reverse primer	cDNA target	Forward primer	Reverse primer
IGFBP2	GGTGGCAAGCATCACCTT	ACCTGGTCCAGTTCCTGTTG	E6	ACTGCAATGTTTCAGGACCCA	TCAGGACACAGTGGCTTTT
GM-CSF	TCTCAGAAATGTTTGACCTCCA	GCCCTTGAGCTTGGTGAG	E7	CAACTGATCTCTACTGTTATGAGCAA	CCAGCTGGACCATCTATTTCA
IGFBP1	AATGGATTTTATCACAGCAGACAG	GGTAGACGCACCAGCAGAGT	HDAC1	CGGTGCTGGACATATGAGAC	TGGTCCAAAGTATTCAAAGTAGTCA
IGFBP3	AACGCTAGTGCCGTCAGC	CGGTCTTCCTCCGACTCAC	HDAC2	CAGATCGTGTAATGACGGTATCA	CCTTTTCCAGCACCAATATCC
IGFBP4	CCTCTACATCATCCCCATCC	GGTCCACACACCAGCACTT	HDAC3	GACCTATGACAGGACTGATGAGG	GAACTCATTGGGTGCCTCTG
IGFBP5	CTACCGCGAGCAAGTCAAG	GTCTCCTCGGCCATCTCA	HDAC4	GTGGTAGAGCTGGTCTTCAAGG	GACCACAGCAAAGCCATTC
IGFBP6	TGACCATCGAGGCTTCTACC	CATCCGATCCACACACCA	HDAC5	CTGAATACCACACCCTGCTCT	CAAGGCAGCACAGCATACAT
IGFBP7	ACTGGCTGGGTGCTGGTA	TGGATGCATGGCACTCATA	HDAC6	AGTTCACCTTCGACCAGGAC	GCCAGAACCTACCCTGCTC
RPLPO	ATCAACGGGTACAAACGAGTC	CAGATGGATCAGCCAAGAAGG	HDAC7	CTCCAGCAGCACCCTCAG	CCAGAGGAAGCAGCACAGT
IGF1R	AAAAACCTTCGCCTCATCCT	TGGTTGTCGAGGACGTAGAA	HDAC8	GAATGTTGACCAGGGAGCAC	CTTCCGCTTAAAACCGTTCC
IGF2R	GCCTGTGTTCCTTCTCCAGT	AGGCCAGTCAGGTCGTACTC	HDAC9	GGCTCAGCTTCAGGAGCATA	CTTCCTGTTCTTGCCTCTGC
INS-RA	CACAACGTGGTTTTCGTCCC	TTTCCGAGATGGCCTAGGGT	HDAC10	TGGGAAGCTCCTGTACCTCTT	GGCTGGAGTGGCTGCTATAC
INS-RB	GTTTTCGTCCCCAGGCCATC	AAGTGTTGGGGAAAGCTGCC	HDAC11	TGTCTACAACCGCCACATCT	CATCCTCTGTGCCCCACT
ADAM17	CCTTTCTGCGAGAGGGAAC	CACCTTGCAGGAGTTGTCAGT	NCOR1	CGCTGATGAGGATGTGGAT	CAGTGAAGGCTTTGAGTCCA
			NCOR2	GTGTACCCGCTGCTGTACC	CTGGTGTTGCCTGGAGACTT
