# Supplementary material for: HPV16 Down-Regulates the Insulin-Like Growth Factor Binding Protein 2 to Promote Epithelial Invasion in Organotypic Cultures
Source: PLoS Pathog. 2015 Jun 24;11(6):e1004988. doi: 10.1371/journal.ppat.1004988 (PMC4479471; doi:10.1371/journal.ppat.1004988)
Supplement: S2 Table — (PDF) [file ppat.1004988.s010.pdf]

Supplemental Table 2: CHIP-qPCR primers used in this study

DNA region	Forward primer	Reverse primer
**IGFBP2 locus**		
-110.5kb	TGCCCTTTGAAGTGTGGTGT	CTGAAGTCAACAGCAGCCTTG
-60.2kb	GCCACATCAAAGCGAGTTCC	CGCCAGCCTAGTTCCTTCTC
-37.2kb	GCACATGGGGAAACTTCTGC	TCTCTGGAAGTCTGCCATGC
-17.7kb	CCAGGATCAAGTCGGATGGG	TGGGCATAGCAGTTCCAAGG
TSS	GGAAGAAGCGGAGGAGGC	CCCACTCTCGGCAGCATG
+856bp	ATGGGGAGAGGAAAGGCATC	CAGCAAATCCCTCCTCCAGA
+26.5kb	CCTGTTCTTGGGGCTCTCAG	TGCCACACTGACATTCCCTC
+32.8kb	TGCATGGGGTCTGATTGTCT	CTTTGCAGCAACTCAGAGGG
Non specific CCND1	TGCCACACACCAGTGACTTT	ACAGCCAGAAGCTCCAAAAA

Supplemental references:

1. Biernacka KM, Uzoh CC, Zeng L, *et al.* Hyperglycaemia-induced chemoresistance of prostate cancer cells due to IGFBP2. Endocr Relat Cancer 2013;20(5):741-51.
